# Evaluating the Return in Ecosystem Services from Investment in Public Land Acquisitions

**DOI:** 10.1371/journal.pone.0062202

**Published:** 2013-06-11

**Authors:** Kent Kovacs, Stephen Polasky, Erik Nelson, Bonnie L. Keeler, Derric Pennington, Andrew J. Plantinga, Steven J. Taff

**Affiliations:** 1 Department of Agricultural Economics and Agribusiness, University of Arkansas, Fayetteville, Arkansas, United States of America; 2 Department of Applied Economics, University of Minnesota, Saint Paul, Minnesota, United States of America; 3 Department of Economics, Bowdoin College, Brunswick, Maine, United States of America; 4 Institute on the Environment, University of Minnesota, Saint Paul, Minnesota, United States of America; 5 World Wildlife Fund, Washington, DC, United States of America; 6 Department of Agricultural and Resource Economics, Oregon State University, Corvallis, Oregon, United States of America; Stockholm University, Sweden

## Abstract

We evaluate the return on investment (ROI) from public land conservation in the state of Minnesota, USA. We use a spatially-explicit modeling tool, the Integrated Valuation of Ecosystem Services and Tradeoffs (InVEST), to estimate how changes in land use and land cover (LULC), including public land acquisitions for conservation, influence the joint provision and value of multiple ecosystem services. We calculate the ROI of a public conservation acquisition as the ratio of the present value of ecosystem services generated by the conservation to the cost of the conservation. For the land scenarios analyzed, carbon sequestration services generated the greatest benefits followed by water quality improvements and recreation opportunities. We found ROI values ranged from 0.21 to 5.28 depending on assumptions about future land use change, service values, and discount rate. Our study suggests conservation is a good investment as long as investments are targeted to areas with low land costs and high service values.

## Introduction

The rapid decline of natural ecosystems around the world has spurred governments, non-governmental organizations (NGOs), and citizens to acquire some of the remaining natural areas for conservation in order to sustain their unique sets of benefits and intrinsic values. However, not all conservation acquisitions create more in societal benefits than social opportunity costs. We estimate the flow of ecosystem services from twenty years of land acquisitions by Minnesota Department of Natural Resources (MNDNR). Our research will help governments, NGOs, and citizens make more cost-effective conservation decisions in the future. For example, in 2008, Minnesota voters passed the Clean Water, Land and Legacy Amendment (Legacy Amendment). The tax will raise an estimated $171 million annually for conservation in Minnesota [1]. Our research can be used to make the acquisitions under this program and other land conservation initiatives more efficient.

The ecosystem services we consider include carbon sequestration, water quality improvement from phosphorous reduction, changes to timber harvest, and changes to consumer surplus from changes in outdoor recreation opportunities. To calculate the benefit of public land acquisitions for conservation from 1989 to 2008 in Minnesota we calculate the level of annual ecosystem services for the Minnesota landscape in 1992, 2022, and 2052 when there is conservation and then counterfactually when there is not conservation. At each point in time, we compare the difference in the provision of ecosystems services from the landscape with and without conservation. We compare the monetary benefit of this conservation to its cost, including initial land acquisition and restoration costs, to find the rate of return on conservation investment (ROI). Along with the acquisitions' ROI, we also consider the impact the acquisitions had on changes in breeding bird habitat quality. Habitat and its associated wildlife (e.g. birds, mammals, and insects) provide ecosystem services (e.g. bio-control, pollination, recreation) that people value. However, unlike the aforementioned ecosystem services, we do not place a monetary value on changes in habitat quality because this is an index of numerous services, and therefore is evaluated separately. We include habitat quality as an indicator in our analysis because governments base acquisition decisions on indicators of ecosystem services as well as habitat quality.

Prior conservation ROI analyses have used biodiversity conservation as the only societal benefit metric. Studies of the relationship between a biodiversity benefit (usually measured as plant and animal species richness) and conservation investment find that return on investment, compared to other conservation prioritization strategies, yields the greatest number of species protected per investment dollar spent (e.g. [2], [3], [4], [5], [6], [7]). The study of conservation in Argentine grasslands in [5] illustrates an inefficient conservation strategy. That analysis shows that a goal of conserving a certain percentage of a country leads to undesirable accumulation of areas with low conservation benefit or requires large sums of investment. Given realistic budgets, an ROI approach to grassland conservation is found to be superior to other conservation strategies [5]. The ROI approach has also been applied to several US landscapes as well. For example, a ROI framework has been developed for the Kona district in Hawaii to guide investment for protection of native forest birds and understory plants [2]. A ROI analysis over the coterminous United States finds that an ROI approach that considers land threatened with conversion and habitat that contains numerous species protects the most species per dollar spent [7].

We make several major contributions to the existing ROI literature. First, the ROI literature typically only considers biophysical returns to conservation while we are considering the monetary return to public investment in conservation. Second, we consider the effect of spatially-explicit LULC change outside of protected areas on ROI. While the ROI literature does consider development within potential conservation sites (e.g., [4], [8]), the literature rarely considers the biological or ecosystem service impact of LULC change in areas surrounding the conservation site. Our econometric model of LULC change and landscape-scale ecosystem service models allow us to predict spatially-explicit LULC change outside of recently acquired protected areas and quantify the impact of this LULC change on the social objective. Third, our econometric model of LULC change also allows us to create counterfactual scenarios that describe what might happen to land in the future if it were not protected. While much of the ROI literature selects sites for conservation based on threat of development (e.g., [7]), it does not explicitly simulate what would happen to the land if not protected and the effect of this alternative scenario on ecosystem service value and species conservation. A better representation of the ramifications of not selecting a site for conservation, and not simply assuming the land will be developed will improve our understanding of the value of purchasing lands for conservation.

### Ecosystem service modeling

We use the InVEST model (Integrated Valuation of Ecosystem Services and Tradeoffs; [9], http://invest.ecoinformatics.org/) along with some other basic analyses to calculate the provision and value of ecosystems services from public land acquisitions for conservation. InVEST allows its user to evaluate the provision of multiple ecosystem services under scenarios of LULC change across a landscape of the modelers choosing. InVEST uses ecological production functions that are explained by LULC pattern and other ecological parameters to determine the biophysical provision (e.g. tons of carbon sequestered) and monetary value (e.g., net present value of sequestered carbon) of ecosystem services produced on an evolving landscape.

A number of papers have used InVEST to quantify ecosystem services produced on a landscape and their economic value. InVEST was used in [10] to compare scores for multiple ecosystem services and biodiversity for stakeholder defined scenarios of LULC change in the Willamette Basin. In [11], InVEST was used to evaluate ecosystem services for actual and alternative land-use change scenarios from Minnesota. Most recently, InVEST is used in [1] to explore the degree of alignment between ecosystem services and biodiversity conservation strategies in Minnesota. By applying InVEST's water quality model over the entire state and not just a region of the state and complementing InVEST with a recreational model, we provide a more comprehensive estimate of ROI from ecosystem services than previous work.

### Site selection with risk of development

The challenge of how best to select undeveloped sites for conservation acquisition has been a pressing issue considered by ecologists and economists for a long time [12], [13], [14]. Economists introduced heterogeneity in land costs to the site selection problem [15] and in more recent years, conservation scientists and economists have incorporated risk of site development [1], [16], [17], [18]. By incorporating risk of development into the problem the likelihood of spending scarce resources to protect land that is not in danger of future development is avoided. For example, [15], which does not consider the risk of conversion, and [16], which does consider the risk of conversion, provide contrary priority rankings for site selection despite analyzing similar data sets. A difference in conservation priorities emerges because land costs and the likelihood of future land use conversion are positively correlated [19].

Calculating expected rates of undeveloped land conversion in the absence of protection can be difficult. Some studies (e.g., [7]) assume immediate historical rates in an area will continue into the near future. Otherwise, economists have built spatially-explicit econometric models of LULC change in order to predict expected changes on the landscape. Economic models of LULC change considering ecological processes are challenged by issues of scale. Socioeconomic variables are collected for administrative units, but ecological processes such as species dispersal or the flow of water across a landscape typically operate at much finer scales, including plot or parcel level scales. Despite the mismatch in the spatial grain of the data and model, several parcel-level LULC change models have been estimated with coarser data [20], [21]. Alternatively, parcel-level econometric models can incorporate greater spatial detail by capturing neighborhood effects [19], [22], [23]. However, these latter models do not incorporate explanatory variables that vary at the regional levels, such as crop and timber prices.

With regard to our LULC change modeling approach, the closest work to this analysis is [21]. In this study the authors integrate fine scale landscape data with US county-level data on net monetary returns to LULC. We use similar data and approaches to create LULC transition probabilities in each Minnesota county. However, unlike [21], we need to allocate expected LULC change at the county level onto a parcel-level map in a coherent manner in order to run the InVEST models. Therefore, we allocate expected county-level change to the 30m parcel level with a LULC suitability map constructed by estimating a statistical relationship between landscape features and LULC pattern in Minnesota.

The rest of the paper is structured as follows. We begin by summarizing the model we use to estimate public conservation's ROI. Then we describe the data and methods for spatially forecasting LULC change across Minnesota out to 2052 with and without 1989 to 2008 public conservation in the third section. The models for ecosystem service provision and value are presented in the fourth section. The fifth section summarizes the results of our research. We conclude with a discussion of the findings and the issues that require further research.

## Overview of the Return on Conservation Investment Calculations

**Figure 1:**
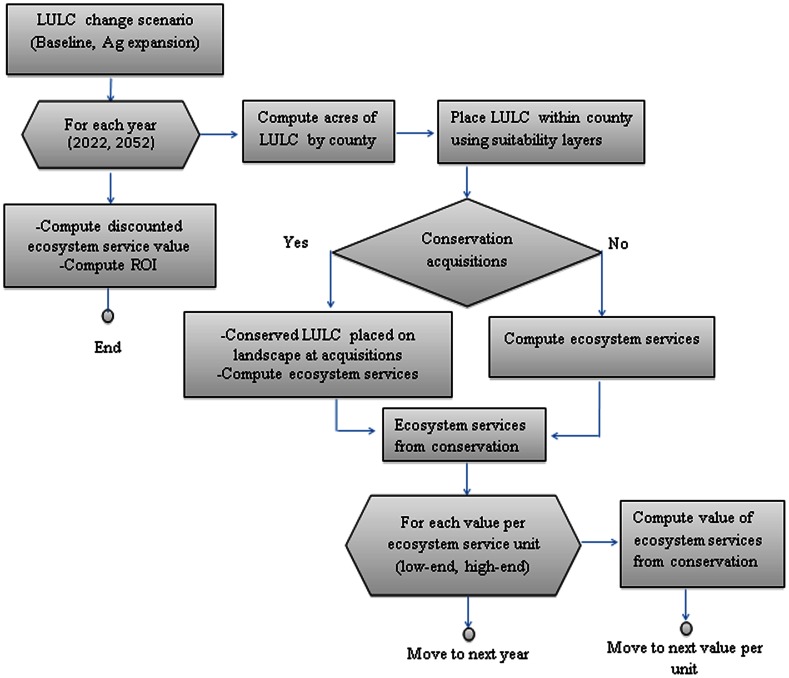
Flow chart of the calculation of the return on investment (ROI) from post-1988 public conservation acquisitions.

We create a model to compute the return on public conservation investment in the state of Minnesota. The model simulates ecosystem service benefits from 1989 to 2052 given state-wide public conservation that took place from 1989 to 2008 (Figure 1). The model consists of two loops, one embedded in the other. The outer loop projects LULC in each Minnesota county in year *t*  = 2022 and 2052 given a LULC change scenario and assuming 1989 to 2008 public acquisitions for conservation *did not occur*. Expected LULC distribution at the county level at *t* = 2022 and 2052 is spatially allocated to the parcel level with 30-meter parcel suitability layers for urban and agricultural development. This collection of maps represents LULC in Minnesota in the future without 1989 to 2008 public acquisitions for conservation. An alternative set of future Minnesota LULC maps is formed by placing the natural LULC associated with the 1989 to 2008 public acquisitions on the first set of maps. This second set of maps represents LULC in Minnesota in the future with 1989 to 2008 public acquisitions for conservation. Then the InVEST tool and the recreation model calculates the provision of ecosystem services on the year *t* landscape with and without post-1988 conservation. The change in ecosystem service provision across the state due to the post-1988 public conservation as of year *t* is then computed.

The inner loop of the model calculates the present monetary value of the *additional* ecosystem services generated up to 2052 on a Minnesota landscape that includes 1989 to 2008 (hereinafter called post-1988) public conservation. We use a representative range of per-unit ecosystem service values or prices when calculating the monetary value of the additional ecosystem services. At this stage we also calculate the economic cost of post-1988 public conservation. Finally, we compute the ROI generated by post-1988 public conservation acquisitions. We consider two LULC change scenarios, so this model is run twice, once for each scenario.

## Description of Methods and Data for Forecasting and Spatially Allocating LULC Change

### Public land conservation

**Figure 2:**
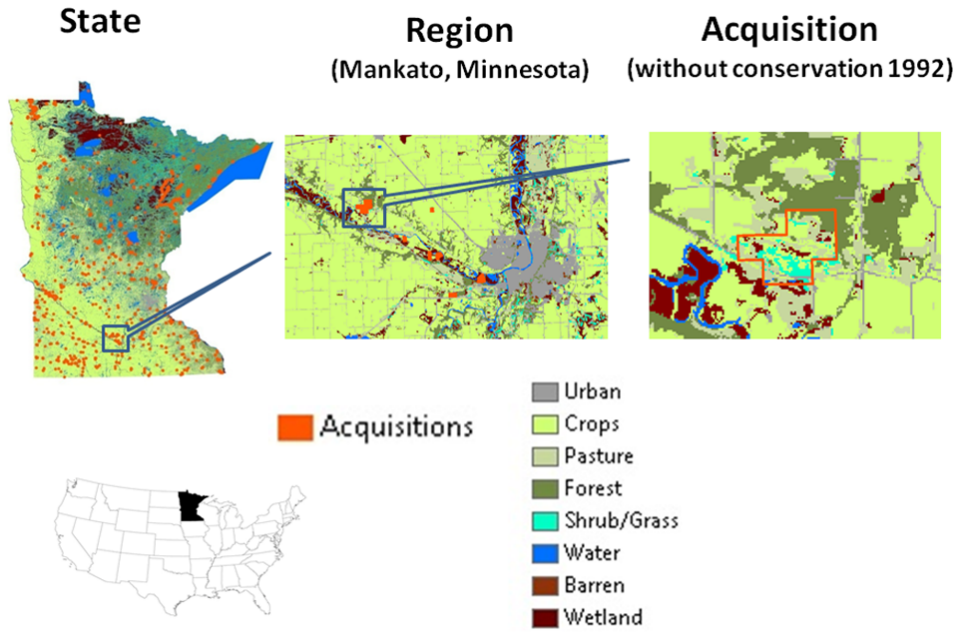
Location of the 1989–2008 acquisitions (i.e., post-1988 acquisitions) and the 1992 land cover without any post-1988 acquisitions.

The MNDNR has geographic information systems (GIS) data of fee-title land acquisitions for conservation from 1989 to 2008 (Figure 2). In our dataset acquisitions less than forty acres are excluded because the location of the parcels on the landscape cannot be accurately determined. This leaves 123,966 acres of land across 680 parcels acquired by the MNDNR from 1989 to 2008 for conservation. The set of acquired parcels span nine MNDNR administrative categories (Table 1). Parcels acquired from 1989 to 2008 often were additions to existing protected areas. Most of acquired land was for wildlife management areas, but state parks, scientific and natural areas, and trails and waterways were also added. Wildlife management areas are not strictly managed for natural cover, and a significant amount of agriculture can occur on these parcels. We make the assumption that the agriculture on these parcels converts to natural cover and thus potentially overestimate the service provision on acquired land.

**Table 1:** Acquisitions by MNDNR administrative classification from 1989 to 2008.

Administrative classification	Acres	Real expenditure (2010$) per acre
Aquatic management area	1,945	5,472
Trails and waterways	16,688	406
Off-highway vehicle	1,710	366
Scientific and natural area	13,654	2,818
State forest	4,455	1,355
State park	13,131	2,815
State recreation area	1,214	1,265
Wild and scenic river	121	770
Wildlife management area	71,047	1,684
Total	123,966	–

We measure the cost of public conservation as the market value of the acquired land plus its restoration cost. The market value data are spatially-explicit; we have land values for every 1989 to 2008 MNDNR fee-title land acquisition. On average, the statewide land value of MNDNR acquisitions from 1989 to 2008 was $1720 per acre [24]. Aquatic management areas tended to be most expensive acquisition ($5472 per acre on average). Scientific and natural areas ($2818 per acre on average) and state parks ($2815 per acre on average) were the next most expensive categories. Government farm payments for cropland are included in the original market value dataset. However, these are transfers and not economic costs from society's point of views and we remove government farm payments from the value of acquired cropland in our analysis. Restoration costs are estimated at the state-level only. On average, the present value of the cost of switching from private to conserved land across the state was $3001 per acre from 1989 to 2008 [25].

### Scenarios of future land use

In this study we run our simulation model for two LULC change scenarios: a baseline and an agricultural expansion scenario. For each scenario we determine the amount of each LULC type in each county in 2022 and 2052 and then spatially allocate any change at a parcel level on the collection of maps *without* post-1988 conservation. We create 2022 and 2052 scenario maps *with* post 1988 public land conservation by placing the appropriate conserved areas on the simulated 2022 and 2052 maps. Land within a conserved area cannot be developed for urban or agricultural use. LULC in an acquisition area not already in a natural state is restored to the natural LULC that is the most common in a five mile radius around the acquisition.

The year of 2052 is chosen as an endpoint of our LULC change scenario to balance the uncertainties of projecting LULC change too far into the future and the goal of estimating the majority of the stream of discounted ecosystem service benefits from the conservation. A midpoint between 1992 and 2052 is chosen in recognition that LULC change is dynamic. However, we avoid more than one time step to keep the analysis of LULC change and ecosystem service provision tractable.

#### Baseline land use change

We create two sets of future Minnesota maps with and without post 1988 public conservation; one where LULC change across the state is expected to proceed as it did in the late 1990s (baseline) and one where crop prices are expected to be higher (agricultural expansion). The baseline projection of LULC change is extrapolated from an econometric model that is estimated with USDA Natural Resources Inventory (NRI) data collected in the 1980s and 1990s [20], [26], [27]. The NRI data reports land use for 844,000 sampled private land plots throughout the United States [28]. The exact location of the NRI plots are not revealed for privacy reasons, however, county location and plot land capability class (LCC) are available. LCC indicates the plot's capability for crop production. LCC is measured on a 1 to 8 integer scale where lower numbers indicate better crop production capability [29]. NRI data more recent than 1997 are not publically available with the full details on county location and plot characteristics. The information from the 1980s and 1990s is sufficient to estimate land use change probabilities for every county and LCC combination.

Our baseline landscape is created by using the output of an econometric model that is estimated with a nested logit specification over NRI data from 1992 and 1997 [20],
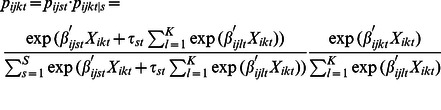
(1)where 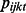 is the probability that plot *i* changes from land use *j* to land use *k* between 1992 and 1997, 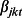 is a vector of parameters associated with the *j*-to-*k* transition, and 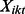 is a vector of independent variables (county net returns [22], land capability measures) for plot *i* in land use *k*. The deterministic component 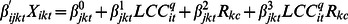
has 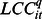
as a dummy variable indicating whether plot *i* has quality *q* at time *t* and 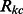 is the level of net returns to use *k* in county *c*. Additional description of the independent variables determining land use change is in Section 2A in Materials S1.

The probability of choosing alternative *k* in 1997 nested in a subgroup *s* given *j* in 1992 can be expressed as the product of the probability 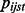 of choosing any of the alternatives grouped within *s*, and the conditional probability 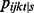 of choosing *k* given the choice of subgroup *s*. The independence of irrelevant alternatives is imposed within but not across the specified subgroups of choices. The NRI data come from a stratified sampling routine that ensure the plots are geographically dispersed.

Estimated model (1) was used to simulate the amount of land that changes from *j* to use *k* in each county for eleven 5-year time steps starting with the 1997 NRI county-level LULC map. This simulation used market prices observed during the 1990s. The simulated change was then used to calculate five year time step county-level LULC transition matrices that give the probability of transitioning from *j* to *k* for all *j*, *k* combinations. [30]. We used the LULC transition matrices from [30] to sequentially obtain the cumulative sum of LULC changes in each Minnesota county from 1992 to 2022 and then from 2022 to 2052 using the 1992 NLCD [31] as our initial map. The Sections S2B-S2C in Materials S1 describe how estimates of 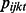 generate the amount LULC change in each county over time and how net returns are endogenous to the amount of land converted over time.

#### Agricultural expansion

This decline in cropland under the baseline may not persist because of demand for corn for ethanol, global population growth, and the demand in developing world countries for the diets of the developed world countries. The real harvest prices in 2011 for corn, wheat, and soybeans, all major crops in Minnesota, are among the highest in the last twenty-five years and are expected to remain high for the next decade [32]. In [30], the estimated model (1) is used in conjunction with higher crop prices to simulate the amount of land that changes from *j* to use *k* in each county for eleven 5-year time steps starting with the 1997 NRI county-level LULC map given systematically high crop prices. We use the eleven 5-year time step transition matrices with higher crop prices created in [30] to simulate county-level LULC change in Minnesota starting in 1992 under higher crop prices. This projection includes a moderate rise in cropland of five percent from 1992 to 2022, and a less than one percent increase from 2022 to 2052. Urban growth is projected to be the same in this scenario as in the baseline. The decline in pasture due to the expansion of cropland and urban is proportional to the changes observed in the baseline projection for these LULC types.

### Projection of future land use to a fine scale landscape

While the future LULC area in each county comes from a simulation of estimated model (1), we use a spatial distribution model to predict where new agriculture and urban uses in 2022 and 2052 will go within a county.

#### Prioritizing the spatial distribution of land use

First, we need to determine which land uses in Minnesota have tended to be converted to urban and agricultural land-uses in the past. We use discrete choice models to correlate past transitions to urban and agriculture land uses as a function of site and regional characteristics. The National Land Cover Database 1992–2001 Land Cover Change Retrofit product (hereafter the NLCD change product; [33]) indicates land use change at a 30m grid resolution within a GIS. For any grid cell (hereinafter referred to as a parcel) that changed land use from 1992 to 2001 we define the million parcels within four kilometers of the parcel as the parcel's region. Then for each parcel that experienced change and its region we create various explanatory variables, including soil quality, proximity measures, percent slope, elevation, and neighboring land uses. Proximity measures include the Euclidean distance from U.S. Census-defined communities and road centerlines of highways. Distance from parcel to the nearest lake is calculated to measure a potential amenity effect on the conversion to urban. More description of the variables used to spatially distributed LULC change is in the section 2D in Materials S1.

We use two statewide probit models to separately estimate the spatial pattern of new urban and agricultural development. The model specification and parameters to be estimated are made explicit:
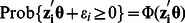
(2)where 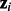 are site and regional characteristics to correlate with the observed transitions of the NLCD change product, 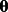 is a parameter vector to be estimated, and 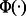 is the cumulative normal distribution. Some of the characteristics that influence the transitions are unobserved, and the unobserved component 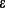 is assumed to be normally distributed. Cells that are developable to agriculture cannot already be in agriculture whereas urban can be developed from agricultural land. We check for spatial autocorrelation in the error matrix of each by calculating Moran's I but find no evidence of this autocorrelation. Hence the estimated models reported in Table 2 are the standard probit models for the full sample.

**Table 2:** Probit Models for Land-Use Change in Minnesota (Baseline Land-Use Category  =  Developable 30-meter grid-cells).

Variable	Urban				Agriculture							
	Marginal Effect	Robust Std. Error		Pr (>|z|)	Marginal Effect		Robust Std. Error			Pr (>|z|)		
Slope	−4.22e–06	2.39e–06		0.07	−7.12e–04		9.39e–05			0		
Elevation	−1.13e–07	4.26e–08		0.01	−4.24e–05		1.47e–06			0		
Distance to Census-defined Community	−9.26e–09	2.10e–09		0	−2.09e–07		3.46e–08			0		
Distance to highways	−1.59e–09	2.43e–09		0.51	−4.21e–07		4.80e–08			0		
Distance to lakes	−3.32e–08	4.39e–09		0	–		–		–			
Distance to urban	−4.12e–06	3.73e–07		0	3.03e–06		5.91e–07		0			
Distance to forest	−4.77e–07	6.99e–08		0	2.81e–06		2.36e–06		0.23			
Distance to agriculture	−6.25e–07	1.29e–07		0	−1.61e–04		1.75e–06		0			
Percent urban												
Half kilometer	2.26e–05	3.02e–06		0	–		–		–			
Three-half kilometer	1.46e–05	1.94e–06		0	–		–		–			
						
Dummy variables for existing LULC (Barren omitted dummy variable)												
Forest	0.04	3.19e–03		0	0.07		2.28e–03	0				
Grassland	0.49	0.02		0	0.98		1.21e–03	0				
Agriculture	0.02	1.67e–03		0	–		–		–			
Soil productivity (Grassland omitted dummy variable)	–	–	–		1.37e–03		1.71e–04	0				
Soil productivity*Forest	–	–	–		−6.31e–03		2.10e–04	0.23				
Number of observations	661,738				310,565							
Log likelihood	−27,056				−53,694							

Note: We estimate separate land-use change models for urban and agriculture because developable grid-cells for urban includes agriculture, though this is not the case for agriculture.

The results from the statewide probit model for urban development finds the conversion to urban is more likely on flat land. Conversion to urban is more likely near Census-defined communities, highways, and other existing urban land cover. The percent of urban within half-mile and one and a half mile both spur urban development. The estimates of the dummy variables of existing LULC types suggest grassland is the most likely to convert, followed by forest, and then agriculture. Proximity to lakes, forests, and agriculture all make the conversion to urban more likely.

We find that conversion to agriculture across the whole state is more likely where there are productive soils. Existing grassland is more likely to convert to agriculture than forest. Steeper slopes and higher elevations make the conversion to agriculture less likely. Proximity to communities and highways makes the conversion to agriculture more likely, while proximity to forest makes conversion less likely.

We then use the predicted model to create a parcel level map of the likelihood or suitability that a parcel, if not already in agriculture or urban, will convert to agriculture and the likelihood that any non-urban parcel initially will convert to urban use.

Our LULC allocation model has several limitations. Protected areas or places incompatible with development for physical or regulatory reasons such as water, urban, and wetlands do not change LULC in the model. Our model omits county-specific land use regulations (e.g. zoning). While land-use regulations are correlated with the spatial pattern of urban and agricultural development, this information is not readily available for the entire state. Finally, the spatial units we use do not spatially align with actual land holding boundaries. This means, for example, that we cannot determine if blocks of parcels transitioned to urban and agriculture uses because the parcels belong to the same owner or if there are spatial interactions among neighboring parcel owners.

#### Assignment of land use to the grid-cell landscape

The 1992 parcel-level land use map of Minnesota is derived from the 1992 NLCD [31]. The 1992 NLCD uses 30m resolution satellite image classification based on Landsat Thematic Mapper imagery. The 1992 NLCD has more LULC types than the NRI used in the econometric LULC change model. The NLCD classes are therefore grouped into forest (NLCD classes 41, 42, 43), cropland (61, 82, 83, 84), pasture (81), grassland (51, 71), and urban (21–23, 85). Existing protected areas, including pre-1989 publically conserved parcels, and urban land, along with the water, barren, and wetland classifications (11, 12, 31-33, 91, 92) on the 1992 NLCD do not transition on post 1992 maps. A description of each of the NLCD land cover classes is in Table S1. Figure 2 illustrates the 1992 land cover for the state, a small region near the city of Mankato, and the boundary of a particular acquisition in that region prior to conservation.

The future landscape is filled out in two steps. First we use the 5-year LULC transition matrices from [30] to determine the expected LULC mix in each county in 2022 and 2052. The new urban parcels projected to be built for 2052 are added to the landscape from the previously created 2022 LULC map. Recall we do this twice, once using 5-year LULC transition matrices assuming 1992 to 1997 market conditions (baseline) and another time assuming higher agricultural prices (agricultural expansion scenario). We then spatially allocate expected LULC mixes in each county in a progressive fashion beginning with urban, followed by cropland, then pasture, and either forest or grassland (depending on the pre-settlement vegetation) fills in the rest of the county. The order is based on the expected rents from each land use. We assume the bid of urban developers always exceeds or equals the bid of farmers for cropland; the bid of farmers for cropland always exceeds or equals the bid of ranchers for pasture, and the bid for pasture always exceeds the bid for natural land. Urban land is allocated to the landscape according to an urban development suitability map. The agricultural development suitability map spatially distributes cropland and then pasture. The remaining land is assigned to the natural cover observed in 1992 or if not previously natural cover then assigned to unmanaged forest or grassland according to pre-settlement vegetation [34].

**Figure 3:**
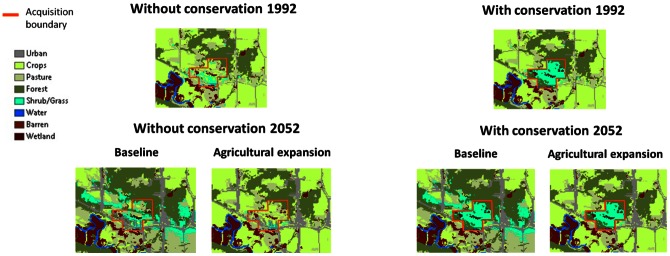
LULC near Mankato, Minnesota in 1992 and 2052 without and with the observed post-1988 public acquisitions for conservation. The baseline and agricultural expansion projections of LULC are shown for 2052.

Figure 3 indicates the LULC within and surrounding a post-1988 acquisition area in 1992 and 2052 with and without post-1988 conservation. The conserved land cover within the acquisition is mostly grassland and shrub but also some forest. This natural cover in the conserved land will persist on the landscape through 2052 although the land cover around the acquisition will change over time. Under both scenarios the publically conserved grassland and pasture would have become cropland without post-1998 public conservation. Table S2 indicates LULC on the acquired land from 1988 to 2008 when there is conservation and when there is no conservation. When there is no conservation, Table S2 indicates the projected LULC on the acquired land for 1992, 2022, and 2052 by land use change scenario.

## Description of the Ecosystem Service Models

We use InVEST to study the change in the provision and value of carbon storage, water quality improvements from phosphorous reduction, and habitat quality and availability on the Minnesota landscape over time. Timber production across the state is estimated with a simple rotation model. The outdoor recreation models were developed by the 2006 Wildlife Habitat Policy Research Program [35] and estimate fishing, hunting, and wildlife viewing use and values on conserved lands. All valuation is discounted to the present value where the present is measure in 2010 dollars.

### Carbon storage and sequestration

The carbon model accounts for carbon (C) stored in the first 30 cm of soil and in above-ground and below-ground biomass. The amount of C stored in each of these pools depends primarily on LULC (e.g., agriculture, forest, grassland) but is also affected by land management (e.g., whether the trees in a forest stand are protected from harvest or are periodically harvested). In addition, we assume that carbon storage is a spatially-independent ecological process: C dynamics in a parcel are not affected by LULC and land management on neighboring parcels.

We assume that by 1992 C in all pools on all private land reaches a level that would not change over time unless the land use or management changed (i.e., the pools are in steady-state or equilibrium on private land as of 1992). We assume storage equilibrium in 1992 because we lack statewide data on LULC ages; information on LULC ages as of 1992 would allow for a more exact estimation of C storage values on the initial Minnesota map. Steady-state C levels for the biomass and soil pools on private and conserved LULC types are listed in Table S2 to S8. In our sensitivity analysis of C storage in biomass we use [36] Materials S1 ‘B tables’ for the low-end of C sequestration rates and data from [37] for the high-end of C sequestration rates.

Given our steady-state assumption, the level of the C in a grid cell only deviates from its initial level if the LULC in the cell changes at least once before 2052. For simplicity, we assume that grid cells that change LULC between 1992 and 2022 and remain privately held over that time period undergo their change in 2007. Therefore, 15 years of C storage flux or sequestration occur on these cells between 1992 and 2022. For the privately-held cells that become MNDNR acquisitions (all MNDNR acquisitions between 1989 and 2008) we assume the LULC change occurs in 1992 and is permanent. Therefore, these cells experience 60 years of C sequestration from 1992 to 2052 or until the cell's C pools reach their subsequent steady-state, whichever comes first.

If a privately held cell changes LULC in 2007 but does not change LULC again before 2052 then the annual sequestration rates that emerged after 2007 continue until a new steady-state is reached or the year 2052. If a privately held cell changes LULC in 2007 and does so again in 2037 (the halfway point between 2022 and 2052) then the annual rates of C pool sequestration in the cell change again in 2037. At this point, sequestration continues in the pools until 2052 or when pool steady-state is reached, whichever comes first. Finally, there are some privately held cells that remain in their 1992 LULC until 2037. In these cells C pool sequestration only begins in 2037. The dynamics of C sequestration for all possible LULC changes are shown in Figures S1 and S2. The C storage across time for the different LULC scenarios without and with post-1988 public conservation is shown in Table S9.

To calculate ROI for a pubic post-1988 conservation acquisition *k* we need to measure the additional C sequestered in the acquisition area compared to the counterfactual where the area is not publically conserved after 1988. The additional annualized C sequestration due to post-1988 acquisition of *k* from 1992 to 2022 is given by 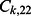,
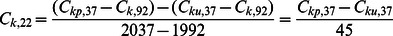
(3)where 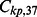 is the C stored in acquisition *k* as of 2037 assuming *k* was acquired by the MNDNR in 1992, 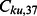 is the C stored in acquisition area *k* as of 2037 assuming *k* was not acquired by the MNDNR in 1992 or any year after, and 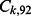 is the C stored in the acquisition area in 1992. C levels in *k* in 1992 are determined by its NLCD cover and in 2037 by *k*'s projected mix of LULC for 2037.

Further, the annualized C sequestration due to post-1988 public conservation of parcel *k* from 2037 to 2052 is given by,
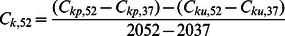
(4)where 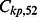 is the C stored in acquisition area *k* as of 2052 and 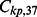 is the C stored in parcel *k* as of 2037 assuming *k* was an area acquired by the DNR in 1992, and 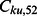 is the C stored in parcel *k* as of 2052 and 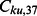 is the C stored in acquisition area *k* as of 2037 assuming *k* was not acquired by the DNR in 1992. C levels in area *k* in the years 2037 and 2052 are determined by *k*'s projected LULC in these years on the respective Minnesota maps. Again, parcels not publically conserved after 1989 do not experience any additional C sequestration under a given scenario.

The annualized sequestration from the C model can be reported as tons of C sequestered or converted to a dollar value by using estimates of the social cost of carbon (SCC), C market prices, or estimates of the cost of C capture and storage [38]. Here we report the value of annualized sequestration using estimates of the SCC from a meta-analysis of peer-reviewed studies [39]. The SCC represents the expected global economic due to an additional ton of carbon dioxide (CO_2_) being emitted to the atmosphere. For the low value for the SCC we used a value of $27.22 per ton of carbon ($7.43 per ton CO_2_) in 2010 dollars, which corresponds to Tol's value for the 33^rd^ percentile from the fitted distribution assuming a 3% discount rate [39]. For the high value for the SCC we used a value of $235.51 per ton of C ($64.23 per ton CO_2_) in 2010 dollars, which corresponds to Tol's value for the 67^th^ percentile from the fitted distribution assuming a 0% discount rate [39]. The European ETS market price for C of 14.91€ per Mg CO_2_ (as of 12 November 2010) translates to $74.87 per ton C, which falls toward the low-end of the SCC range.

### Water quality and value from phosphorous reduction

The land cover transitions modeled in our analysis will impact both the amounts of additional nutrients (in the form of chemical fertilizers applied to agricultural lands) and the ability of lands to retain excess nutrients en route to downstream water bodies. For example, natural vegetation established on conserved parcels improves regional water quality relative to urban or agricultural land uses through both reduced export and improved nutrient retention. Here we focus on phosphorus pollution in surface waters, which is a leading cause of water impairment in the Midwest [40]. We used the InVEST water quality model to estimate the annual nutrient retention service provided by each modeled landscape. Unlike the carbon sequestration model, the water quality model is spatially dependent: the quality of a water body is a function of LULC and management on all upstream parcels, whether conserved or not.

We ran the water quality model for each of the eight-digit hydrologic unit code (HUC) basins in Minnesota. The InVEST water yield model estimates the expected annual water yield in each grid cell based on climate, geomorphological information, and LULC characteristics. The model assumes that all precipitation not lost to evapotranspiration contributes to the surface water runoff and subsurface flows that constitute the water yield at the watershed outlet.

To estimate nutrient retention across the landscape, the water yield output is combined with data on expected phosphorous loading and the filtering capacities or retention coefficients for each LULC type (see Table S13). Expected excess phosphorous from each parcel is routed downstream based on a digital elevation model, where some of the phosphorous may be filtered or additional phosphorous added, until it flows into a water body. This model structure makes results sensitive to the spatial pattern of land use in each basin. In particular, stream buffers of perennial vegetation may effectively filter phosphorous before it reaches a stream. Once nutrients reach a water body the model assumes no additional retention or removal before delivery to the mouth of the watershed.

We convert the annual loadings of phosphorous at the mouth of each eight-digit HUC into monetary values using the results from a recent meta-analysis that summarizes the willingness to pay (WTP) for improved water quality of lakes and rivers in the United States [41]. Following the guidelines in [42] we adapted parameters in the WTP function to reflect the baseline water quality, median household income, and the number household affected by the water quality of the watershed. The model estimates WTP as a function of changes in water quality relative to baseline conditions, with water quality described according to the Resources for the Future (RFF) water quality ladder. The RFF water quality ladder links changes in water uses (drinking, boating, swimming, and fishing) to variations in biophysical characteristics (dissolved oxygen, turbidity, pH) and uses a qualitative point system to represent changes in the value of uses that correspond to changing water quality [43].

To establish baseline water quality for each HUC basin, we obtained statewide data on lake trophic state index (TSI, [44]) from the Minnesota Pollution Control Agency (MPCA 2012, Heiskary, personal communication). We then mapped average TSI values for lakes within each HUC basin to the RFF water quality ladder. Based on consultation with local water quality experts, we assumed that a 50% reduction in phosphorous loading relates to a two-point increase along the RFF water quality ladder. Combining these water quality parameters with the WTP function in [41], we generated estimates of annual WTP for the 50% reduction from $24.97 to $44.72 per household in 2011 constant dollars. The values were prorated to the percent change in phosphorous loadings modeled by InVEST; for example, for a WTP value of $10 per household for a 50% reduction, a 1% reduction in phosphorous loadings was prorated to $0.20.

We compared these results to WTP values from [45] specific to phosphorus reductions in the Minnesota River Basin. Mathews et al. reported an average value of $140 per household per year in 1997 dollars, or $183.71 in 2010 constant dollars for a 40% reduction in phosphorous loadings. The WTP estimates from [45] were 4–7 times greater than WTP values from the meta-analysis in [41], therefore we used each estimate as an upper and lower bound on WTP for modeled phosphorus reductions.

After prorating WTP values to reflect modeled phosphorus reduction we adjusted household WTP values based on estimates of weighted visitation and the weight household income of the visitors to each basin. Weighting was based on the number of households in 1992 and population projections for 2022 and 2052 [46] as well as assumptions about how visitation to recreational lakes declines as a function of distance from population centers (Section 6B in Materials S1).

### Habitat extent and quality

The InVEST habitat model accounts for the spatial extent and quality of habitat for a targeted conservation objective (e.g., forest birds, amphibians, etc.). Here we consider two objectives, forest breeding birds and grassland breeding birds. Maps of LULC are transformed into maps of habitat for a class of birds by defining what LULC counts as habitat for birds in that class. Habitat quality in a grid cell is a function of the LULC in the grid cell, the LULC in surrounding grid cells, and the sensitivity of the habitat in the grid cell to the threats posed by the surrounding LULC (like the water quality model, the habitat model is spatially dependent). Each LULC type is given a habitat suitability score of 0 to 1 depending on the class of birds being considered. For example, grassland breeding birds may prefer native prairie habitat above all other habitat types (habitat suitability  = 1), but will also make use of a managed hayfield (habitat suitability  = 0.5). See Table S10 for the definition of habitat suitability and quality across LULC types for each bird class.

The habitat quality score in a grid cell can be modified by LULC in surrounding grid cells. We consider sources of degradation such as human modified LULC types (e.g., urban, agriculture, roads) that cause edge effects [47]. Edge effects refer to changes in the biological and physical conditions that occur at a patch boundary and within adjacent patches (e.g., facilitating entry of predators, competitors, invasive species, toxic chemicals and other pollutants). The sensitivity of each habitat type to degradation is based on general principles of landscape ecology and conservation biology (e.g., [48], [49]) and is specific to each bird class. See Table S10 and S11 for the sensitivity scores and the influence of threats determined from the literature and expert knowledge.

We generate a habitat quality score for both bird classes on each of the four modeled Minnesota landscapes. Because of the influence of adjacent patches on quality scores, the spatial pattern of LULC as well as the overall amount of habitat determines the landscape habitat quality score. Habitat quality scores should be interpreted as ordinal scores with higher scores indicating landscapes more favorable to supporting breeding bird abundance. The landscape habitat quality score cannot be interpreted as a specific prediction of species persistence on the landscape or other direct measure of species conservation in the same way that the output of the carbon model is an estimate of the actual carbon stored on the landscape. The InVEST habitat model does not convert habitat quality measures into monetary values.

### Value of timber production

We use data from [20], [26], [27] to estimate annual net returns to timber forestry for the year 1992, 2022, and 2052. Timber harvest is assumed to occur on private forest and conserved forest land designated as State Forest by the MNDNR but not on other land (Table S12). We multiply working forest acreage in a post-1988 acquisition in a given year by the county's per acre net return to forestry in that year to calculate the total value of forestry in the acquisition. Estimated returns to forestry in a county are based on the assumption that all state forests are managed on an optimal, even-age rotation basis to produce saw timber.

For the landscape without post-1988 conservation, the value of timber harvest is assumed to be capitalized into the purchase price of the land, which is reflected in the land value component of the acquisition costs. We assume the timber is sold into national and international markets so that timber prices do not change with changes in harvest volumes and that inputs to timber harvest are able to respond to alternative production patterns such that per unit production costs remain constant.

### Outdoor recreation and value

The post-1988 public conservation acquisitions increased the land available for outdoor recreation in Minnesota. These purchases in turn increased the visitation and the value of recreation. We use a suite of recreation models developed for the 2006 Wildlife Habitat Policy Research Program [35] to evaluate the number of new fishing, hunting, or wildlife viewing visitor days associated with the acquisitions over the course of a year.

The suite of visitation models were originally estimated from a sample of National Wildlife Refuges with data on visits per activity, refuge size, refuge natural features (lakes, rivers, and oceans), and per-capita income and population within a 60-mile radius of the particular refuge [50]. For this model we assume that all post-1988 acquired land resembles National Wildlife Refuges in terms of visitation rates. The wildlife-watching and fishing visitation models' explanatory variables include acquisition size and per-capita income and county population surrounding the acquisition (Table S14 and Table S16). The hunting visitation model's explanatory variables include acquisition size and the presence of water (Table S15). Scientific and natural areas do not allow fishing, hunting, and wildlife-watching visitation so we do not estimate visitor days for these areas. Also, hunting is not allowed at state parks.

The number of new visitor days to the acquisitions depends on the surrounding amount of existing public land. We assume a new acquisition will attract more visitors if there is little public land in the region. The acreage of pre-existing public land is obtained from the protected areas database [51]. We use high and low-end estimates for visitation based on the amount of nearby public land that could act as an alternative visitation site. The low-end estimate for visitation assumes the acquired land is similar to all the existing public land. The high-end estimate for visitation assumes the acquired land is similar to half of the existing public land.

The monetary values for outdoor recreation are the average consumer surplus values for a day of fishing, hunting and wildlife-viewing, which are $40, $42, and $47 respectively [35]; Table S17). Loomis [52] assembles a database of consumer surplus values that is up to date in terms of the studies available as of the beginning of 2007. The daily hunting surplus is based on the average of 192 estimates from 21 studies of big game, small game, and migratory bird hunting value per day in the Northeast. The daily fishing surplus is the average of 58 estimates from 14 studies of cold water fishing value per day in the Northeast. The daily wildlife-viewing surplus is the average of 81 estimates from 9 studies of wildlife-viewing value per day in the Northeast. Estimates of the annual visiting days per activity across all post-1988 acquisitions are multiplied by the daily value of the activity to arrive at a statewide annual value per activity on post-1988 acquisitions. The annual value of fishing, hunting, and wildlife-viewing on post-1988 acquisitions is summed to calculate the total annual value of recreation on Minnesota's recently acquired public lands.

## Results

We determine the provision of annual ecosystem services for the Minnesota landscape in 1992, 2022, and 2052 when there is conservation and then counterfactually when there is not conservation. At each point in time, comparing the difference in the flow of ecosystems services from the landscape with and without conservation evaluated at the value per service unit (i.e. low or high) allows us to compute the present value of returns from conservation. For timber harvest and outdoor recreation, we calculate the value for the landscape with the conservation only because the value for timber harvest and outdoor recreation on the private land are assumed to be capitalized into the value of the land.

Table 3 indicates the increase in the provision of ecosystem services with conservation under both scenarios (the baseline and agricultural expansion). Carbon sequestration increases 39,000 to 71,000 metric tons annually, depending on the LULC change scenario and the assumed above-ground biomass productivity. Phosphorous loadings to water bodies decline in 2022 and 2052 by less than a quarter of a percent due to the acquisition of protected areas. Some basins exhibit greater improvements in water quality with post-1998 public conservation due to a greater restoration and avoided development in those basins and the adjoining upstream basins. Habitat quality and extent for breeding forest and grassland birds as of 2022 and 2052 also improves due to post-1998 public conservation by a quarter percent or less under the baseline scenario and by a half a percent under the agricultural expansion scenario. Results for the water and habitat quality models reflect the inherently spatial nature of ecological processes and the importance of considering surrounding landscape-level processes when managing for these services. The annual visitor days for outdoor recreation as of 2022 and 2052 increase by 18,000 to 41,000 due to public conservation, depending on the assumed abundance of public land around the acquisitions.

**Table 3:** Annual biophysical change in ecosystem services with versus without the post–1988 acquisitions from the Minnesota landscape under the baseline and agricultural expansion LULC change scenarios.

			Baseline		Agricultural expansion	
Ecosystem services	Sensitivity	1992	2022	2052	2022	2052
Carbon sequestration (metric tons of C)	High: [36]	68,565	67,816	67,622	70,585	70,692
Carbon sequestration (metric tons of C)	Low: [37] “Materials S1 B Tables”	40,228	39,585	39,472	41,226	41,289
Water pollution reduction: phosphorus		0.15%	0.11%	0.11%	0.21%	0.23%
Change in birds biodiversity measure	Forest birds	0.23%	0.23%	0.21%	0.39%	0.43%
Change in birds biodiversity measure	Grassland birds	0.26%	0.21%	0.19%	0.41%	0.45%
Timber production (harvested acres)		3,161	3,161	3,161	3,161	3,161
Outdoor recreation (visitor days)	High: Scarce public land	33,837	36,759	40,677	36,759	40,677
Outdoor recreation (visitor days)	Low: Abundant public land	18,704	20,322	22,496	20,322	22,496

Table 4 shows the annual value of the ecosystem services from the acquisitions by year for the baseline and the agricultural expansion scenarios. Future annual values are discounted to the present to make the returns from ecosystem services comparable through time. We use a real discount rate of 2% because the acquisitions are a public good. Many environmental economists argue that public goods should be discounted at the market rate of return for risk-free financial assets even if the public good has the riskiness of financial assets such as corporate stocks [53]. The present value of the complete 60-year stream of annual ecosystem services from 1992 to 2052 is computed by summing the present value of annual stream of ecosystem services from the 1992 landscape for 15 years (1992 to 2007), then the present value of annual stream of ecosystem services from the 2022 landscape for 30 years (2007 to 2037), and the present value of annual stream of ecosystem services from the 2052 landscape for the remaining period of ownership by the state (2037 to perpetuity). In the baseline and agricultural expansion scenarios, the annual value is higher in 1992 than 2022 and 2052 because of the influence of discounting. Also, in the baseline, LULC change reduces cropland, and this makes the annual return from conservation less in 2022 and 2052 than in 1992.

**Table 4:** Annual value of ecosystem services (thousands 2010 $) from post-1988 conservation for 1992, 2022, and 2052 for the baseline and the agricultural expansion LULC change scenarios.

			Baseline			Agricultural expansion		
Ecosystem services	Value per unit of service (2010$)	1992	2022	2052	Present value	2022	2052	Present value
Carbon sequestration	$235.36 per tC	16,137	12,585	6,928	773,074	13,100	7,242	791,200
Carbon sequestration	$27.22 per tC	1,095	850	468	52,294	885	489	53,523
Water pollution reduction: phosphorus^a^	$0–8.62 per household depending on basin	1,900	1,554	1,103	99,368	1,489	1,491	107,346
Water pollution reduction: phosphorus^a^	$0–1.57 per household depending on basin	400	295	208	19,672	279	281	21,095
Timber production	-$0.67–5 per acre depending on county	10	7	5	479	7	5	479
Outdoor recreation	$40–47 per visitor day depending on activity	1,700	1,345	821	83,981	1,345	821	83,891
Outdoor recreation	$40–47 per visitor day depending on activity	910	744	454	45,801	744	454	45,801
Sum value of all services	High	19,747	15,491	8,857	956,902	15,941	9,559	982,916
	Low	2,415	1,896	1,135	118,246	1,915	1,229	120,898

The present value of stream of annual ecosystem services for each LULC change scenario is calculated.

a The per household value of a percent reduction in phosphorous is from [45] at the high end and from [41] at the low-end.

The largest economic returns are associated with carbon sequestration, followed by water quality improvement from phosphorus reduction and the visitor days from outdoor recreation. Each of these services generates an annual value of over $800,000 at the high-end value per unit and over $200,000 at the low-end value per unit (Table 4). Timber harvest generates an annual value of only $10,000 because less than four percent of the acquired land is state forest. Outdoor recreation value ranks highly because this occurs on all acres of acquired land except for the Scientific and Natural Areas. On the other hand, carbon sequestration and water quality improvements only occur if there is restoration or avoided development. Aggregating over all services, the annual return exceeds eight million on the high-end and exceeds a million on the low-end, and the influence of discounting erodes the present value of returns from the ecosystem service in 2022 and 2052.

**Figure 4:**
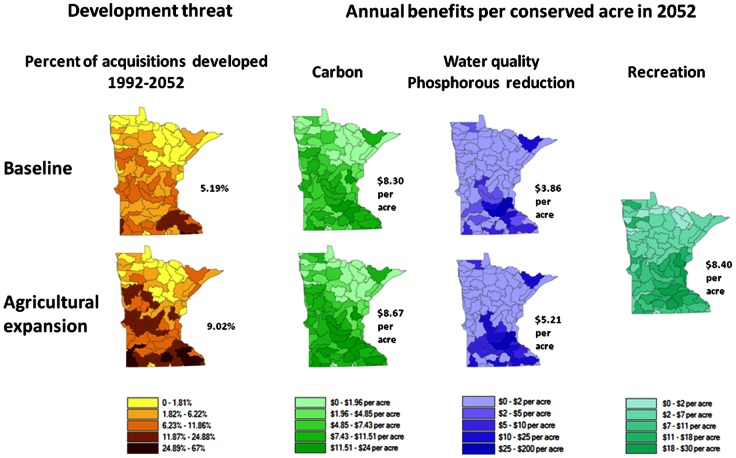
Development threat represented by the percent of post-1988 acquisition area converted to agriculture or urban assuming no conservation from 1992 to 2052. The annual benefit per acre (2010$) of acquisitions in 2052 are shown by 8-digit watershed for carbon, reduction of phosphorous loadings, and recreation for the baseline and agricultural expansion scenarios. The value estimates assume the low end of service values. The numbers by the side of each map indicate the state average.

**Figure 5:**
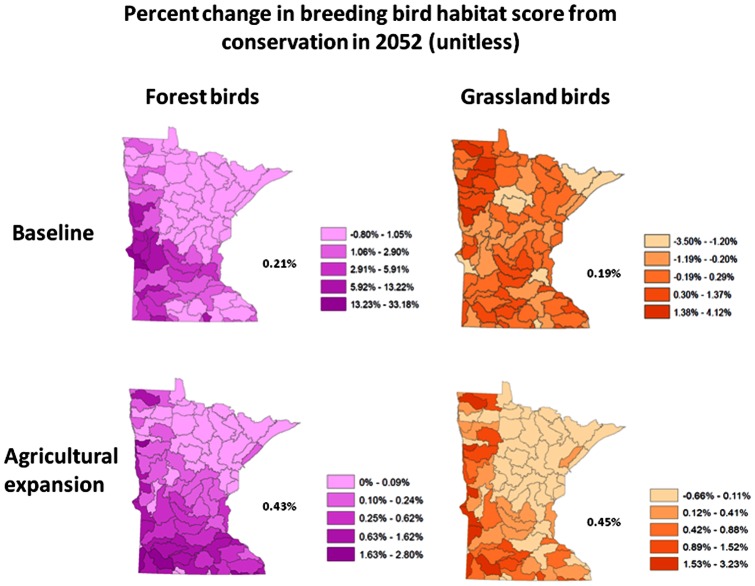
1992 to 2052 change in the forest and grassland breeding bird habitat scores for the baseline and agricultural expansion scenarios. The numbers by the side of each map indicate the state average.

The maps of the development threat in Figure 4 represent the percent of the acquisitions developed with urban or agriculture LULC in the baseline and agricultural expansion scenarios *without* post-1998 public conservation. Also, the annual return per acre in ecosystem services for 2052 from the acquisitions is shown using the low-end service value. The development threat is largest in the southern and central regions of the state where the agricultural and population centers are located. The carbon value per acre is the highest in the southeast because of the reforestation of riparian areas. The value of the phosphorous reductions per acre is largest in watersheds connected to rivers that flow through large populations, the watersheds where there is the greater development threat, and where low levels of phosphorous pollution exist (i.e. even a small reduction in phosphorous can mean a large percentage decrease in phosphorous). The return per acre from recreation is highest where many people live, the per capita income is large, and the existing amount of public land in the watershed is low. Figure 5 shows the forest and grassland breeding bird habitat quality increases where there is a reduced development threat and suitable surrounding habitat. The forest bird habitat improves more than the grassland bird habitat in the baseline, and the vice versa for the agricultural expansion scenario. In the baseline, the loss of forest and grassland occurs from the development of pasture across the state while in the agricultural expansion scenario the loss of forest and grassland occurs mostly because of an expansion of cropland.

The present value of benefits per acre for all ecosystem services, costs per acre, ROI, and the pay-back period for both LULC change scenarios is shown in Table 5. The present value ranges from $950 per acre at the low-end value per service unit for the baseline scenario to $7950 per acre for the high-end value per service unit for the agricultural expansion scenario. In the case of Minnesota public land acquisitions, the development threat embodied by the LULC change scenario affects the benefits per acre less than the sensitivity for the low and high-end returns per service unit. The average cost per acre of an acquisition, including the cost of restoration and the value of the land, is $3010. The ROI is defined as the ratio of the present value of the benefits per acre to the acquisition cost per acre. A value of one or above indicates the state receives a return from ecosystem services equivalent or greater than the investment in the land. The low-end of the ROI ranges from 0.32 to 0.33 and the high-end ranges from 2.57 to 2.63. The difference in the low and high-end of the ROI comes from the value per metric ton for carbon and the productivity of biomass, the value of water quality, and the amount of surrounding public land. A sensitivity of the ROI to the discount rate indicates that a 1% discount rate on the high-end value per service unit increases ROI to 5.14–5.28 and a 3% discount rate on the low-end value per service unit decreases ROI to 0.21–0.22. At the high-end of the ROI, the state pays back the cost of investment in the acquisitions in 25 years.

**Table 5:** Present value of benefits and costs per acre (2010 $), return to investment, and the pay-back period.

Return on investment in ecosystem services			Baseline	Agricultural expansion
Present value of benefits per acre	High		7,720	7,930
Present value of benefits per acre	Low		954	978
Costs per acre			3,001	3,001
	High	1% discount rate	5.14	5.28
Return on investment	High	2% discount rate	2.57	2.64
	Low	2% discount rate	0.32	0.33
	Low	3% discount rate	0.21	0.22
Years to pay back investment	High		25.4	24.7
Years to pay back investment	Low		–	–

**Figure 6:**
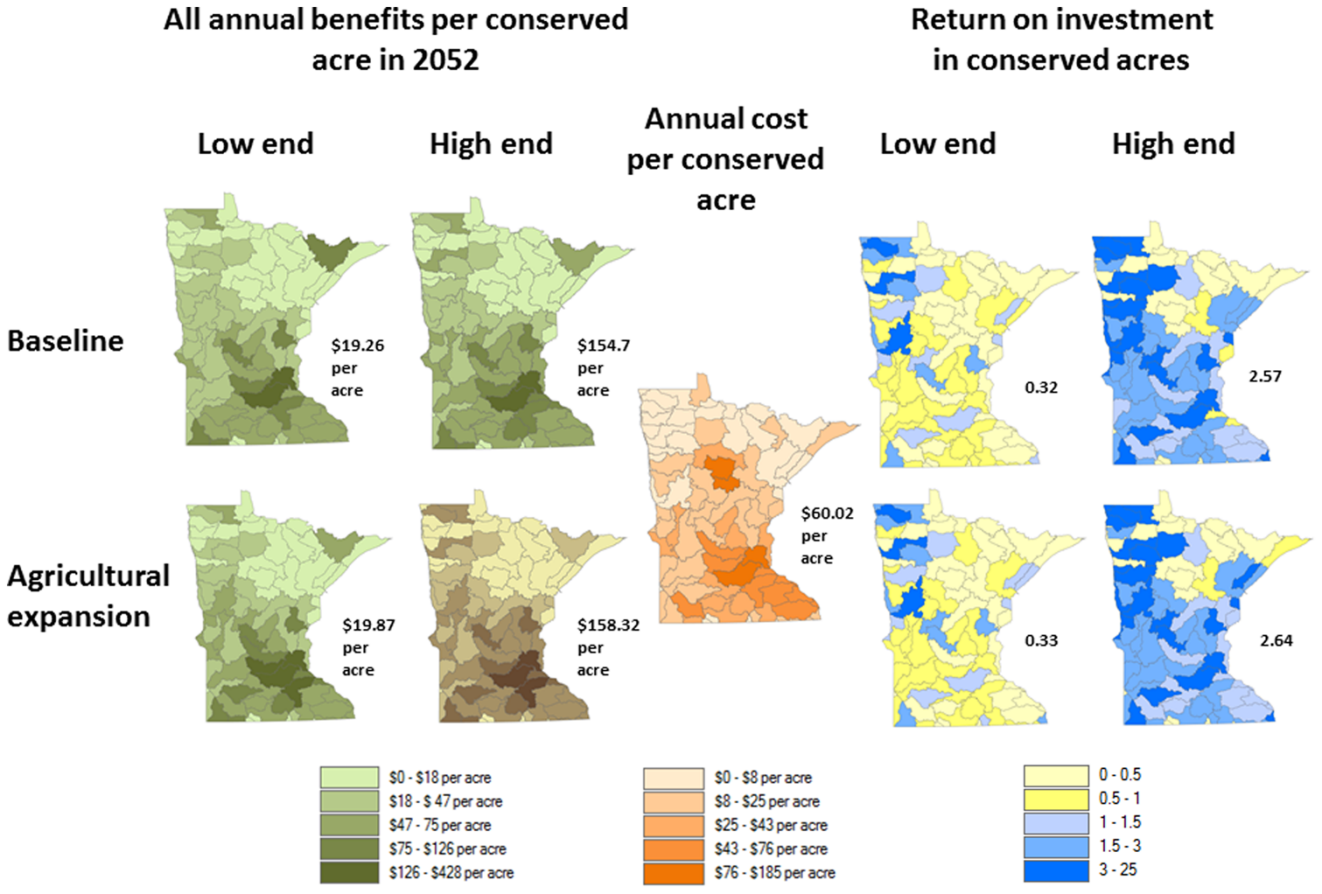
The range in benefits per acre (2010$) for all the ecosystem services (species scores not included), the annualized costs per acre, and the range in return on investment from the post-1988 public acquisitions under the baseline and agricultural expansion scenarios. The numbers by the side of each map indicate the state average.

**Figure 7:**
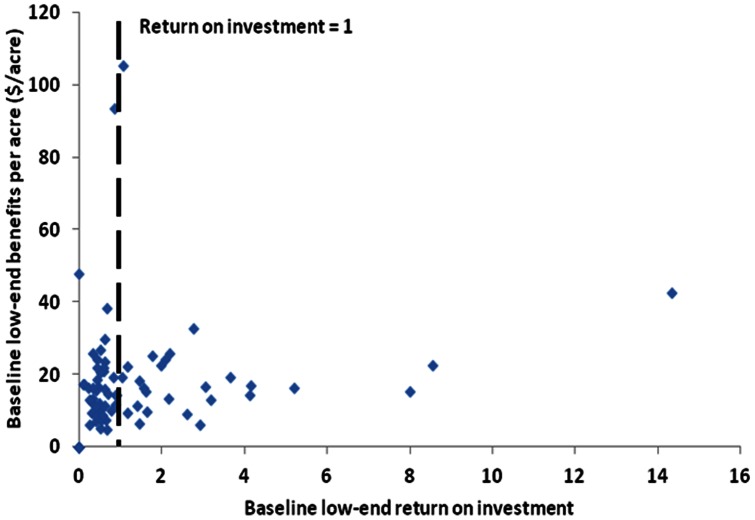
Tradeoff between the benefits and the return on investment from post 1988 public conservation as of 2052 for each 8-digit watershed under the baseline LULC change scenario. Here we use the low-end of service values. The dashed vertical line separates the watershed with a return on investment below one from the watershed with a return on investment above one.

Annual benefits and costs per acre, and the ROI in 2052 are shown by eight-digit watershed in Figure 6. The annual benefits per acre at the low end is $19 to $20 per acre and at the high end is $154 to $158 per acre for the baseline and agricultural expansion scenarios. The annual benefit per acre of acquisition is highest in the south and the west where the agricultural and population centers are, but the costs per acre are also highest in these regions. Putting annual benefits and costs per acre together, the ROI is found to be the highest in the west and the north. The costs per acre are relatively higher in the south due to higher opportunity costs. To examine further why the ROI is higher in some watersheds of the state, we consider the tradeoff between the benefits per acre and the ROI from conservation in Figure 7. The figure illustrates that watersheds with the highest ROI are associated with the low costs per acre rather than high benefits per acre. Restoration and thus carbon sequestration occurs on nearly all the acquisitions. Also, the value per metric ton of carbon is the same everywhere, and carbon value is a large proportion of the aggregate annual benefits. As a consequence, the benefits per acre across watersheds are within a narrow range, and the watersheds with low land costs are those with the highest ROI.

**Figure 8:**
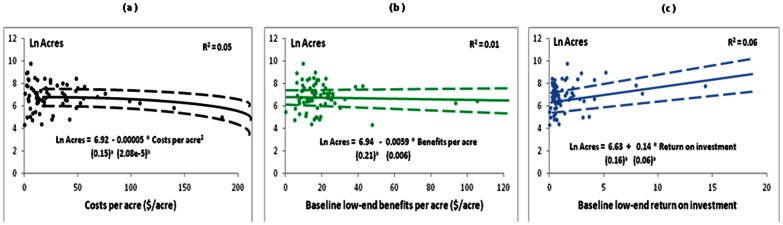
Natural log of the post-1988 publically acquired acres for each 8-digit watershed as explained by the annual (a) cost per acre, and the baseline LULC (b) benefits per acre in 2052 (in 2010 $) and (c) return on investment. Here we use the low-end of service values. Solid line represents the best fitting model, dashed lines represent ±1 standard error (SE). Robust standard errors shown in parentheses. ^a^ indicates indicates significance at the 10% level. The best fit in panel (a) based on the residual sum of squares criterion is a non-linear quadratic.

Three approaches for acquiring land are targeting based the least cost per acre, the highest benefit per acre, or the highest ROI. The study of how the MNDNR made past acquisitions can help improve the decisions in the future. Figure 8 indicates three scatter plots to consider which approach most closely matches the acquisitions by the MNDNR. The amount of acres purchased in an eight-digit watershed is plotted against the cost per acre, the estimated benefits per acre, and the ROI. The relationship with the cost per acre is negative and statistically significant while the relationship with benefits per acre is not statistically significant. The ROI relationship is positive and statistically significant, and ROI has the strongest fit according to a least sum of squares criterion. The evidence is that targeting by the state is based on the least cost per acre, and since ROI is constructed from the opportunity cost of acquisitions, the targeting is also correlated with the highest ROI.

## Discussion

We evaluate the ROI from conservation acquisitions by the state of Minnesota from 1989 to 2008 by considering the joint provision of multiple ecosystem services and biodiversity, the cost of the acquisition and restoration, and the development threat. Our results indicate the return from ecosystem services exceeds the cost of investment in conservation for both the baseline and agriculture expansion scenarios when the high-end service values are used. Given the types of ecosystem services evaluated and the value per unit of service, our case study of Minnesota indicates public land acquisitions with a higher ROI typically have low costs and average benefits. The post-1988 acquisitions with the lowest costs are typically the better investments even though the avoided development and associated ecosystem service benefits are below average. A statistically significant inverse relationship is found between the amount of conserved land in a watershed and the cost of the acquired land. Empirically, this conservation strategy is consistent with the highest return on investment because benefits per acre do not vary much across the study area.

The spatial pattern of LULC change outside acquisition areas influences how effectively the acquisitions reduce phosphorous and improve the habitat quality. This makes modeling LULC change for the entire landscape important for the assessment of the ROI from protected areas. We observe changes in habitat quality do not always align with the ecosystem service flows, and thus further study is needed about how to balance these alternative conservation priorities. Most acquisitions have very similar ROIs under both scenarios of LULC change when holding the per unit value of ecosystem services constant. However, the ROIs of acquisitions are often quite different when the full range of per unit of service values is used. This uncertainty in what the value of a unit of service is affects the targeting of land for conservation. Larger non-market values for the reduction of phosphorous in the Mississippi River make acquisitions close to the river and upstream of the Twin Cities metropolitan region a higher priority. The considerable range in the estimates of the SCC confounds the choice of acquisitions for primarily carbon and the choice of acquisitions for carbon and water quality. An exploration of uncertainty using distributions of service values rather than high and low bounds on values would indicate the expected ROI rather than a range. Further, values for non-market goods and services are functions that depend on the level of the provision of various goods and services rather than constants in a given year as we assume.

An increasing number of studies now consider multiple ecosystem services in addition to biodiversity for evaluating landscape change and conservation planning decisions. Nelson et al. [54], using data from the Willamette Basin, predict the provision of carbon sequestration and species conservation for policies that offer payments for conservation. Nelson et al. [10], also using data from the Willamette Basin, compare scores for multiple services and biodiversity for stakeholder defined scenarios of LULC change. In Minnesota, Polasky et al. [11] evaluate ecosystem services, biodiversity conservation, and the returns to landowners for actual land-use change and a suite of alternative land-use change scenarios. However, these studies have not used ecosystem services and biodiversity to evaluate the ROI from actual past conservation decisions. Polasky et al. [1] use Minnesota data to find the optimal ROI from carbon sequestration and phosphorous reduction associated with optimal land purchases for an actual future conservation budget. The purchases, which represent the optimal choices that Minnesota could make according to the data available, yield a return on investment for the high end value per service unit of $2 to $3 per dollar invested.

Our analysis provides evidence of the ROI from multiple ecosystem service when accounting for the spatial dependencies of LULC important for phosphorous reduction and biodiversity. There are a host of additional factors that can be considered. One important issue not considered is land market feedbacks between conservation strategies and land prices [55], which then might drive land-use decisions on unprotected land. More research investigating spatial externalities in the spatial arrangement of agricultural and urban development within counties is needed. While we found a positive return on investment for the past level of investment by the MNDNR, we did not attempt to solve for the optimal investment that would maximize social net benefits. The largest beneficiaries of the acquisitions are those potentially affected by climate change or those who have the means to travel long distances for recreation. The values per service unit from a less wealthy segment of society could change the priority of conservation purchases. We do not estimate flood damage reduction, pollination potential, air quality improvements, or the aesthetic value associated with proximity to open space. To the extent these ecosystem services have positive value we underestimate the full return from conservation. Consideration of management and conservation practices, such as fertilizer application rates and riparian buffers, may provide additional insights of how to improve environmental performance on a landscape. Finally, consideration of the spatial interactions, where the benefit of acquiring a parcel depends on where earlier parcels are acquired, and dynamic transition paths, such as the time path of accumulation of carbon with forest maturation rather than analysis of steady-state conditions, are important lines of further inquiry.

## Supporting Information

Figure S1 **Soil carbon sequestration dynamics in a grid cell.** (**A**) A private grid cell begins 1992 in LULC *i*, transitions to LULC *j* in 2007, and to LULC *k* in 2037. (**B**) A private grid cell begins 1992 in LULC *i* and transitions to LULC *j* in 2007. (**C**) A private grid cell begins in LULC *i* and transitions to LULC *j* in 2037. (**D**) A private grid cell begins 1992 in LULC *i* and transitions immediately to conserved LULC *m* in 1992. Because the soil reaches its new SS storage level in 50 years the soil will stop sequestering carbon in 2042.
(TIF)

Figure S2 **Biomass carbon sequestration dynamics in a grid cell. We assume a LULC transition clears all of the previous accumulated biomass in a cell and its associated carbon.** (**A**) A private grid cell begins 1992 in LULC *i*, transitions to LULC *j* in 2007, and to LULC *k* in 2037. (**B**) A private grid cell begins 1992 in LULC *i* and transitions to LULC *j* in 2007. (**C**) A private grid cell begins 1992 in LULC *i* and transitions to LULC *k* in 2037. (**D**) A private grid cell begins 1992 in LULC *i* and transitions immediately to conserved LULC *m*. (E) A private grid cell begins 1992 in LULC *i* and transitions immediately to conserved LULC *m* in 1992.
(TIF)

Materials S1 **Materials in support of the land use change and ecosystem service modeling.**
(DOCX)

Table S1 **LULC class definitions from the definitions of the grouped classes of the NLCD 1992 used in the maps for Minnesota (from** http://www.mrlc.gov/nlcd92_leg.php **).**
(DOCX)

Table S2 **LULC on the acquisition for the maps with acquisitions, and the maps without acquisitions in 1992, 2022, and 2052 for the baseline and agricultural expansion scenarios.**
(DOCX)

Table S3 **Distribution of metric tons of stored soil organic carbon (SOC) per hectare within the 30 centimeters of the soil profile by LULC type and county (estimated from** [**4**] **). We assume crop SOC is 75% of natural LULC SOC estimates.**
(DOCX)

Table S4 **Metric tons of stored biomass carbon per hectare by non-forest LULC type.**
(DOCX)

Table S5 **Metric tons of stored biomass carbon per hectare on private forests and rotation length (in years) using “B” tables in** [**15**] **.**
(DOCX)

Table S6 **Metric tons of stored biomass carbon per hectare on private forests and rotation length (in years) using estimates in** [**16**] **.**
(DOCX)

Table S7 **Metric tons of stored biomass carbon per hectare in 2052 on forests conserved in 1992 assuming the 1992 LULC was a private forest using “B” tables in** [**15**] **.**
(DOCX)

Table S8 **Metric tons of stored biomass carbon per hectare in 2052 on forests conserved in 1992 assuming the 1992 LULC was a private non-forest using “B” tables in** [**15**] **.**
(DOCX)

Table S9 **Metric tons of stored soil and biomass carbon in 1992 and the carbon stored with and without conservation in 2037 and 2052 for each LULC change scenario.**
(DOCX)

Table S10 **Sensitivity to degradation sources and habitat suitability weights each LULC type for breeding bird biodiversity. Higher numbers indicate more sensitivity or more suitable habitat.**
(DOCX)

Table S11 **Weights and effective distances for degradation sources used in the habitat quality model.**
(DOCX)

Table S12 **Average per acre net returns to managed forestry from** [**3**] **,** [**19**] **,** [**20**] **(all values are expressed in 1992 dollars; 1992  = 100).**
(DOCX)

Table S13 **Estimates for nutrient loading, evapotranspiration, rooting depth, available water capacity, and vegetation filtering.**
(DOCX)

Table S14 **Coefficient estimates for the model of non-consumptive (wildlife viewing) visits.**
(DOCX)

Table S15 **Coefficient estimates for the model of total hunting visits.**
(DOCX)

Table S16 **Coefficient estimates for the model of total freshwater fishing visits.**
(DOCX)

Table S17 **Average values per day for hunting, fishing, and wildlife viewing.**
(DOCX)
