# Supplementary figures and images for: Evaluating the Return in Ecosystem Services from Investment in Public Land Acquisitions
Source: PLoS One. 2013 Jun 11;8(6):e62202. doi: 10.1371/journal.pone.0062202 (PMC3679083; doi:10.1371/journal.pone.0062202)

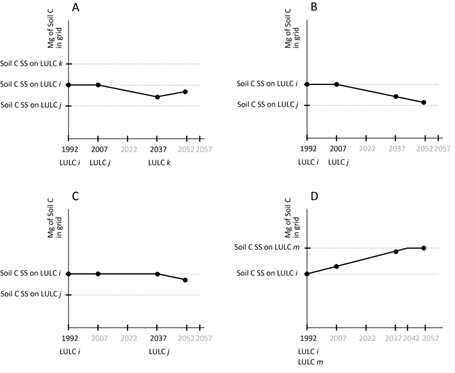

Supplement: Figure S1 — Soil carbon sequestration dynamics in a grid cell. (A) A private grid cell begins 1992 in LULC i, transitions to LULC j in 2007, and to LULC k in 2037. (B) A private grid cell begins 1992 in LULC i and transitions to LULC j in 2007. (C) A private grid cell begins in LULC i and transitions to LULC j in 2037. (D) A private grid cell begins 1992 in LULC i and transitions immediately to conserved LULC m in 1992. Because the soil reaches its new SS storage level in 50 years the soil will stop sequestering carbon in 2042. (TIF) [file pone.0062202.s001.tif]

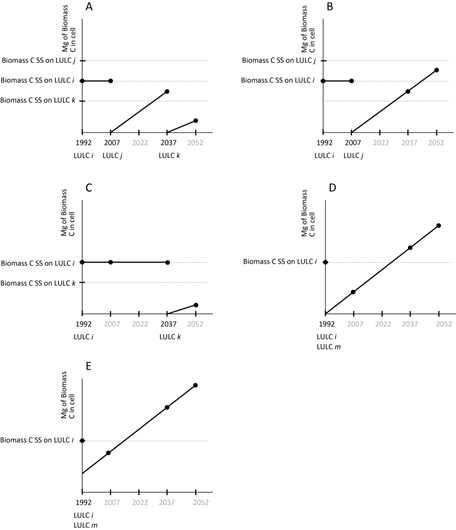

Supplement: Figure S2 — Biomass carbon sequestration dynamics in a grid cell. We assume a LULC transition clears all of the previous accumulated biomass in a cell and its associated carbon. (A) A private grid cell begins 1992 in LULC i, transitions to LULC j in 2007, and to LULC k in 2037. (B) A private grid cell begins 1992 in LULC i and transitions to LULC j in 2007. (C) A private grid cell begins 1992 in LULC i and transitions to LULC k in 2037. (D) A private grid cell begins 1992 in LULC i and transitions immediately to conserved LULC m. (E) A private grid cell begins 1992 in LULC i and transitions immediately to conserved LULC m in 1992. (TIF) [file pone.0062202.s002.tif]
