# Supplementary material for: Evaluating the Return in Ecosystem Services from Investment in Public Land Acquisitions
Source: PLoS One. 2013 Jun 11;8(6):e62202. doi: 10.1371/journal.pone.0062202 (PMC3679083; doi:10.1371/journal.pone.0062202)
Supplement: Materials S1 — Materials in support of the land use change and ecosystem service modeling. (DOCX) [file pone.0062202.s003.docx]

**1. Land use / land cover (LULC) maps**

We create ten maps of land use / land cover (LULC) in Minnesota at the grid cell level (cell size = 30 x 30 m). Two maps have the LULC pattern of the 1992 NLCD outside of the acquisitions, and the LULC on the acquisitions of one 1992 map has only native natural cover (with acquisitions). Four maps have a predicted LULC pattern for 2022 based on the projections of the econometric model, two maps for the baseline scenario (with and without acquisitions) and two maps for the agricultural expansion scenario (with and without acquisitions). There are also four maps for 2052 based on the projections of the econometric model, two maps for the baseline scenario (with and without acquisitions) and two maps for the agricultural expansion scenario (with and without acquisitions). The econometric model for the projection of the LULC change is described in the main text.

On each LULC map each grid cell is assigned a one-digit classification (1 to 8) of LULC. The NLCD classes are grouped into forest (4) (NLCD classes 41, 42, 43), cropland (2) (NLCD classes 61, 82, 83, 84), pasture (3) (NLCD class 81), grassland/shrub (5) (NLCD classes 51, 71), and urban (1) (NLCD classes 21-23, 85). Existing protected areas and urban land, along with the water (6), barren (7), and wetland (8) classifications (NLCD classes 11, 12, 31-33, 91, 92) in the 1992 NLCD do not transition. The definitions of the LULC categories [1] are given in Table S1.

*1A. LULC on the acquisitions*

A summary of LULC on the acquisitions for the maps with acquisitions and the maps without acquisitions in 1992, 2022, and 2052 for the baseline and agricultural expansion scenarios is summarized in Table S2. Area is measured in acres.

The LULC on the acquisition lands for the ‘with acquisiton’ maps do not have urban, cropland, or pasture by construction of the maps. The 1992 map without acquisitons includes developed and natural land, and the developed land is mostly in agriculture and pasture, and much less urban while the natural land is mostly forest and wetland with some grassland. Comparsion of the ‘with acquisition’ LULC and the 1992 map without acquisitons indicates the restored developed land becomes mostly grassland/shrub and a third becomes forest sinc e the developed acquisition land mostly agriculture and pasture built over historically grassland and shrub.

Th e LULC on the ‘without acquistion’ maps in 2022 and 2052 have alternate trends depending on the baseline and agricultural expansion scenarios. Urban expands equivaltently in both of the LULC change scenarios, but the grassland/shrub and forest land cover steadily rises in the baseline scenario but steadily fall in the agricultural expansion scenario. The rise of natural cover in the baseline scenario or the fall in the agricultural expansion scenario is low in comparison to the increase in the natural cover from the restoration of the developed land. Thus, the difference in the provision of ecosystem services from the acquisiton of these lands by comparing the two LULC change scenarios is unlikely to be large.

**2. Scenarios of future land use**

*2A. Description of the independent variables determining land use change*

The county-level net returns to forestry included in the independent variable vector $X_{it}$are measured as the annualized revenues from timber production less management costs. The county-level agricultural net returns included in the independent variable vector $X_{it}$are equal to the weighted average of the annual revenues from crop and pasture production less costs and plus government payments. Returns to urban land in the independent variable vector $X_{it}$are measure the annualized median value of a recently developed parcel used for a single-family home, less the value of structures. Landowners form expectations of future returns based on the average of the annual net returns over the preceding five-year period. Dummy variables for the land capability classes are interacted with county average net returns to scale the returns up or down according to the productivity of the plot.

*2B. Generation of land use change from transistion probabilites*

We define$A_{ijt}$as associated with NRI point *i* in use *j* in time *t*. In the initial period, each point is in one of the land uses as indicated in the NRI data. Given a sequence of transition probabilities, we estimate how much land will be distributed across the land use categories at each time in the future. As in equation (1) of the main text, the probability that land represented by point *i* transitions from use *j* to *k* during the interval beginning at *t* is denoted$P_{ijkt}$.

We express the land at point *i* that converts from use *j* to *k* during the interval beginning in *t* as $TA_{ijkt}=P_{ijkt}\cdot A_{ijt}$. The acres represented by point *i* in use *j* at time *t*+1 are given by,$A_{ijt+1}=\sum_{k} TA_{ijkt}=\sum_{k} P_{ijkt}\cdot A_{ijt}$, reflecting the first-order Markov structure of the model. More details on the land use change model are available in [2], [3].

*2C. Endogenous price effects*

The induced change in land use implies a change in the supply of land-based commodities and services and, hence, changes in related prices and net returns. We model these endogenous price effects for forest, cropland, and urban, and assume that net returns to pasture, range, and CRP remain constant in the simulation. Details on the justification of ignoring the price effects of other land uses are in [3]. Consistent with the model of landowner behavior, supplies of crop and forest commodities are inelastic. Crop and timber yields (per acre) are thus used to translate land-use changes into output changes. After aggregating output changes, corresponding price changes are computed using own-price demand elasticities estimated in previous econometric studies.

The price changes resulting from the land-use changes are used to form new measures of net returns. Using the new net returns, we recalculate the transition probabilities and repeat the procedure. This stage of the simulation ends when the crop and forest net returns have converged (that is, period-to-period changes in price are near zero). The converged net returns are equilibrium values that reflect all of the anticipated supply adjustments in agricultural and forest commodity markets. A second stage of the simulation proceeds by recalculating transition probabilities using the converged net returns for cropland and forests, and the observed net returns for other uses. The sequence of land use transitions is then computed as described in section 2A of supplementary except the net returns remain at their equilibrium values throughout this stage.

*2D. Description of the independent variables to spatially distributed future LULC*

We created dummy variables for the developable LULC types (barren, forest, grassland, and agriculture) from the NLCD change product. We construct a soil productivity value based on non-irrigated yields within sub-county soil regions with the U.S. General Soil Map (STATSGO2) Database (USDA National Resources Conservation Service, <http://soils.usda.gov/survey/geography/statsgo/>).[[1]](#footnote-1) We construct a set of dummy variables to indicate the land use prior to conversion to urban or agriculture to account for differences in conversion cost based on the land cover. Interaction variables of soil quality and the dummy variable for the forest land type control for the effect that productive forest has on the potential for agriculture. Also, we create proximity measures to the 1992 urban, forest, grassland, and agriculture from the NLCD Change Product to examine the effect of neighboring LULC types. Variables for the percent of urban within half and one and a half kilometer radius account for the likely agglomeration of new urban development around existing urban land. A set of dummy variables for each county in the state is also added to control for unobserved county-specific effects. County specific land use regulations (e.g. zoning) are also correlated with the spatial pattern of urban and agricultural development, but this information is prohibitively difficult to obtain for the entire state.

**3. Carbon storage and sequestration**

We assume land-use grid cells, including those in working forests, have attained their LULC biomass and SOC storage steady-state levels or equilibrium as of 1992. Per hectare equilibrium levels for all LULC types and their sources are listed in Tables S3 to S8.

*3A. Soil and biomass carbon dynamics*

Figures S1 and S2 indicate the carbon sequestration dynamics from the possible LULC scenarios with and without the conservation. Table S9 shows the metric tons of stored soil and biomass carbon in 1992 and the carbon stored with and without conservation in 2037 and 2052 for LULC change scenario. Soil carbon is measured at the 30 cm depth. For the calculation of the annualized carbon sequestration with and without conservation, we use the biomass carbon storage based on “B” tables in [15] for the low end and [16] for the high end.

Figure S1 demonstrates the sequestration dynamics in the soil pool. The four panels in the Figure S1 indicate the following. **(A)** A private grid cell begins 1992 in LULC *i*, transitions to LULC *j* in 2007, and to LULC *k* in 2037. The dark line gives soil carbon (C) levels over time. The slope of the line between 2007 and 2037 indicates the annual rate of soil C sequestration across the LULC transition. The slope of this piece of the sequestration curve is equal to soil C steady-state (SS) on LULC *j* less soil C SS on LULC *i* divided by 50 (we assume it takes soil 50 years to reach its new C SS after a change). The slope of the sequestration curve between 2037 and 2052 indicates the annual rate of soil C sequestration across the second LULC transition. The slope is equal to the soil C SS on LULC *k* less soil C in the cell in year 2037 divided by 50. **(B)** A private grid cell begins 1992 in LULC *i* and transitions to LULC *j* in 2007. The slope of the sequestration curve after 2007 indicates the annual rate of carbon soil sequestration across the LULC transition. The slope is equal to soil C SS in LULC *j* less soil C SS on LULC *i* divided by 50. **(C)** A private grid cell begins in LULC *i* and transitions to LULC *j* in 2037. The slope of the sequestration curve after 2037 indicates the annual rate of carbon soil sequestration across the LULC transition. The slope is equal to soil C SS on LULC *j* less soil C SS on LULC *i* divided by 50. **(D)** A private grid cell begins 1992 in LULC *i* and transitions immediately to conserved LULC *m* in 1992. The slope of the sequestration curve indicates the annual rate of carbon soil sequestration across the LULC transition. The slope is equal to soil C SS on LULC *m* less soil C SS on LULC *i* divided by 50. Because the soil reaches its new SS storage level in 50 years the soil will stop sequestering carbon in 2042.

Figure S2 demonstrates the sequestration dynamics in the biomass pool. The five panels in the Figure S2 indicate the following. **(A)** A private grid cell begins 1992 in LULC *i*, transitions to LULC *j* in 2007, and to LULC *k* in 2037. The dark line is the biomass C sequestration curve. The slope of the curve indicates the annual rate of biomass C sequestration across a LULC transition. The slope of the sequestration curve path from 2007 to 2037 is equal to the annual rate of biomass C sequestration on LULC *j*. The slope of the sequestration curve from 2037 to 2052 is equal to the annual rate of biomass C sequestration on LULC *k*. We assume it takes newly established non-forest LULC 50 years to reach its SS C level after a change. Private forest carbon SS age is given by the forest’s rotation length (see the appendix). **(B)** A private grid cell begins 1992 in LULC *i* and transitions to LULC *j* in 2007. The slope of the sequestration curve after 2007 indicates the annual rate of biomass C sequestration on LULC *j* when not in SS. **(C)** A private grid cell begins 1992 in LULC *i* and transitions to LULC *k* in 2037. The dark line gives biomass C levels over time. The slope of the sequestration curve after 2037 indicates the annual rate of biomass C sequestration on LULC *k* when not in SS. **(D)** A private grid cell begins 1992 in LULC *i* and transitions immediately to conserved LULC *m*. This sequestration pattern holds for LULC *i* and *m* transitions where at least one LULC, either *i* or *m*, is not a forest. If *m* is a non-forest then biomass C SS is reached by 2042 in the cell. If *m* is a forest LULC then biomass C SS is not reached by 2052. **(E)** A private grid cell begins 1992 in LULC *i* and transitions immediately to conserved LULC *m* in 1992. This sequestration pattern holds for LULC *i* and *m* transitions where *i* is a private forest and *m* is a public forest. Here we assume a portion of the private forest biomass remains on the land that is transitioning to public forest (given by the y-axis intercept). Biomass C SS is not reached by 2052.

**4. Biodiversity conservation: Habitat extent and quality**

For each species group we assign a habitat suitability score to each LULC type ranging from 0 to 1, with non-habitat scored as 0 and the most suitable habitat scored as 1, with marginal habitat scored in between. For example, grassland songbirds may prefer native prairie habitat above all other habitat types (habitat suitability = 1), but will also make use of a managed hayfield (habitat suitability = 0.5). For this study we scored habitat differently based on its level of state and federal protection. We used the Minnesota Department of Natural Resources GAP data on stewardship for the state: code 1 and 2 are publicly protected lands, code 3 is land under an easement, and code 4 private lands [17]. We assume the habitat quality potential of a LULC increases with the level of protection. See Table S10 for information on habitat suitability scores of LULC types for breeding bird biodiversity.

Second, we evaluate the impact of threats, which can degrade and reduce habitat quality in a grid cell either directly (e.g., habitat loss) or indirectly (e.g., edge effects from habitat fragmentation). Designated threats for this study include urban and agricultural areas, and primary, secondary, and tertiary or light roads. Urban and agriculture areas were quantified directly from the scenario LULC map while roads were evaluated using a statewide road layer [17]. The impact of threats is mediated by three factors.

The first factor we determine is the relative impact of each threat on a habitat grid cell. Because some threats are more damaging for all habitats, we assign a relative impact score to all threats (see Table S11). A threat’s weight, *w_r_*, indicates the relative negative impact of a threat. For example, if urban grid cell has a weight of 1 and road cell a weight of 0.5 then the urban area causes twice the degradation, all else equal.

We assign a threat-mitigating factor represented as the distance between the grid cell and the threat and the impact of the threat across space. If a grid cell is within the assigned impact distance of a particular threat then the grid cell is within the threat’s degradation zone. In general, the severity of a threat on habitat quality decreases as distance from the habitat grid cell to the threat increases, so that grid cells that are proximate to a threat will experience higher degradation or lower habitat quality. We use an exponential distance-decay rate to describe how a threat’s impact diminishes over space. For example, if the maximum distance of a threat is set at 1 km, the impact of the threat will decline by ~ 50% when a habitat pixel is 200 m from the defined threat. The impact of threat *r_y_* on habitat in grid cell *x*, given by *i_rxy_*, is normalized by the maximum effective distance of threat *r*, *d_r_*_max_, and is represented by the following equation,

$$i_{rxy}=\exp\left(-\left(\frac{2.99}{d_{r\max}}\right)d_{xy}\right)$$

where, *d_xy_* is the distance between grid cell *x* and the source of threat *r*, grid cell *y*.

We determine the relative sensitivity of a habitat type in a grid cell to all threats and is the final input used to generate the total degradation level a grid cell. Let *S_jr_*$\in$[0,1] indicate the sensitivity of habitat type *j* to degradation source *r* where values closer to 1 indicate greater sensitivity to a threat. For example, a forest habitat patch may suffer more degradation from an adjacent pasture (more sensitive) than a grassland habitat patch (lower sensitivity). The model assumes the more sensitive a habitat type is to a threat, the more degradation to that habitat will be caused by that degradation source. A habitat’s sensitivity to threats is based on general principles from landscape ecology (e.g., [18]).

Therefore, the total threat level in grid cell *x* with LULC or habitat type *j* is given by *D_xj_*,

$$D_{xj}=\sum_{r=1}^{R} \sum_{y=1}^{Y} w_{r}r_{y}i_{rxy}\beta_{x}S_{jr}$$

where, *y* indexes all grid cells on the landscape (including *x*). If *S_jr_* = 0 then *D_xj_* is not a function of threat *r*.

We calculate the quality of habitat in parcel *x* of LULC *j* by *Q_xj_* where,

$Q_{xj}=H_{j}\left(100-D_{xj}\right)$

Therefore, when *Q_xj_* = 100 the quality of habitat in grid cell x is at its maximum.

We give a habitat quality landscape score for each scenario, which is an aggregate of all grid cell-level habitat quality scores on the landscape under each scenario.

**5. Forestry returns**

Estimated returns to forestry development on the landscape were modeled (see Table S12) using data from [3], [19], [20], which found average per acre county-level net returns to commercial forestry for 1992. We multiplied county *i*’s 1992 per acre net forestry returns by a scenario’s acres of forest on state forest land and then summed across the two values to determine county *i*’s net forestry returns for that scenario.

**6. Water quality and value**

*6A. Water quality and yield models*

The following model descriptions are adapted from [21]. For each scenario we determined water yield and total phosphorous loadings for the Minnesota 8-digit watershed. First, we model water yield, which approximates the absolute annual water yield across the basin, and is calculated as the difference between precipitation and actual evapotranspiration on each grid cell. We used maps of 30-year mean annual precipitation and reference evapotranspiration (adapted from data provided by the Minnesota State Climatology Office), soil depth and plant available water content [22], as well as data on the coefficients of rooting depth [23] and evapotranspiration (adapted from [24]) for each LULC type (See Table S1).

The water yield model is based on the Budyko curve, developed by [25], and annual average precipitation. We determine annual water yield (*Y_jx_*) for each grid cell on the landscape (indexed by *x* = 1,2,…,*X*) as follows:

$$Y_{jx}=\left(1-\frac{AET_{xj}}{P_{x}}\right)\cdot P_{x}$$

where, *AET_xj_* is the annual actual evapotranspiration on grid cell *x* with LULC *j* and *P_x_* is the average annual precipitation on grid cell *x*. The evapotranspiration partition of the water balance, $\frac{AET_{xj}}{P_{x}}$, is an approximation of the Budyko curve [25].

$$\frac{AET_{xj}}{P_{x}}=\frac{1+\omega_{x}R_{xj}}{1+\omega_{x}R_{xj}+\frac{1}{R_{xj}}}$$

where, $R_{xj}$is the Budyko Dryness index on a grid cell *x* with LULC *j*, which is the ratio of potential evapotranspiration to precipitation [26]. $\omega_{x}$ is an annualized ratio of plant accessible water storage to expected precipitation.

$$\omega_{x}=Z\frac{AWC_{x}}{P_{x}}$$

where, *AWC_x_* is the volumetric plant available water content measured in mm and is estimated as the difference between field capacity and wilting point. *AWC_x_* is defined by soil texture and effective soil depth, which establishes the amount of water capacity in the soil that is available for use by a plant. *Z* is the Zhang constant that presents the seasonal rainfall distribution. Finally, with *R_xj_* is calculated by the following,

$$R_{xj}=\frac{k_{xj}\cdot ETo_{x}}{P_{x}}$$

where, *ETo_x_* is the reference evapotranspiration on grid cell *x* and *k_xj_* is the plant evapotranspiration coefficient associated with the LULC *j* on pixel *x*. *ETo_x_* represents an index of climatic demand while *k_xj_* is largely determined by a grid cell’s vegetative characteristics [24].

Second, we determine the quantity of phosphorous retained by each grid cell in the watershed using information on nutrient loadings based on export coefficients and filtering characteristics of each LULC (see Table S13; [27]), the water yield output noted above, and a Digital Elevation Model. Adjusted Loading Value for grid cell *x*, *ALV_x_,* is calculated by the following equation:

$$ALV_{x}=HSS_{x}\cdot pol_{x}$$

where, *pol_x_* is the export coefficient at grid cell *x* and *HSS_x_* is the Hydrologic Sensitivity Score for grid cell *x* and is calculated as:

$$HSS_{x}=\frac{\lambda_{x}}{\bar{\lambda}_{W}}$$

where, is the mean runoff index for the basin, and *λ_x_* is the runoff index for grid cell *x* and is calculated by the following:

$$\lambda_{x}=Log\left(\sum_{U} Y_{U}\right)$$

where, $\sum_{U} Y_{U}$is the sum water yield of all grid cells along the water flow path above and including grid cell x.

Once we determine *ALV_x_*, we then estimate how much of the load is retained by each grid cell downstream of a neighboring cell, as surface runoff moves phosphorous across the landscape and towards the mouth of the watershed. Using a GIS, we model the route of surface water down flow paths as determined by the slope of a grid cell. Each grid cell downstream is allowed to retain phosphorous based on its land-use type. Finally, the model aggregates the phosphorous loading that reaches the stream from each grid cell to determine the total loading for the entire watershed.

*6B. Accounting for the number of household affected by the water quality of a watershed*

The final step in the determination of the value of water quality is the multiplication of the prorated water quality value per household by the number of households affected by the water quality change. The number of households affected by a water quality change depends on where people perform their water based activities. Not all activities occur in the watersheds where the households reside. The water based recreation patterns of households can be used to reapportion the households from where they live to where their water based activities occur. Counting the households in a watershed based on how many do their water based activities there better represents how many households are affected by a water quality in that watershed.

We use three steps to account for the number of households affected by water quality in each watershed. First, to represent visitation patterns for water based activities in Minnesota, a table from a report on the valuation of Iowa lakes [28] determines the average number of trips to lakes taken in 2002 by the residents of nine zones in Iowa. We estimate the distance from each of the nine zones to all of the other zones in Iowa to create a matrix of distance between zones. An ordinary least squares (OLS) regression using White’s correction for heteroskedasticity estimates how quickly trips decline with distance from a place of residence. The estimated relationship is:

$$\theta_{ij}=0.733-0.01M_{ij}+0.0000334M_{ij}^{2}$$

where, $\theta_{ij}$ is the proportion of water based trips spent in watershed *j* by the residents of watershed *i*, and $M_{ij}$ is the miles traveled between watershed *i* and the watershed *j*. We set the proportion equal to zero for miles greater than 123 since this is where the estimated function crosses the horizontal axis from above. The estimated relationship is then transferred to Minnesota to estimate how visitation to lakes in Minnesota is affected by distance from where the households live.

Second, we find the distance from each of the HUC08 watersheds to all of the other eighty-one watersheds. We then estimate how many trips are taken for a representative household in a watershed to all of the other watersheds using the results of the OLS regression from the first step. For each of eighty-one watersheds, we determine the proportion of the trips to each of the surrounding watersheds based on the estimate of the number of trips to each watershed. Third, this weights matrix with the dimensions of eighty-one squared is applied to a vector of the number of households that live in each of the watersheds and the vector of the mean household income of each watershed. The result is the weighted number of households affected by the water quality in each watershed and weighted mean household income of the household affected by the water quality in each watershed.

**7. Recreation visitation and value**

The purpose of the visitor use estimating models for wildlife viewing, hunting, and fishing is to relate MNDNR acquisition visitation to acquisition acres, natural features of the acquisitions (e.g., lakes and rivers), population and income in the surrounding area. We adapt the visitor use estimating models from [29] to determine the visitation to the public lands acquired by MNDNR. We relate the model from [29] to Minnesota acquisitions by accounting for the presence of surrounding public land and excluding the explanatory variable for the presence of an ocean.

*7A. Statistical results of the visitor use estimating models*

The following model descriptions are adapted from [29]. The model coefficient estimates come from a model of visits to National Wildlife Refuges with data from [30]. Loomis et al. [29] determine refuge acres and natural features from brochure and planning documents about the refuges. Per capita income is for the counties surrounding the refuge. County population is based on the population of all counties within a 60 mile radius surrounding the particular refuge. Explanatory variables statistically significant at the 10% level or higher are retained for predicting non-consumptive (wildlife viewing) visits, total hunting visits, and fishing visits to the refuges.

The coefficient estimates for the model of non-consumptive visits is shown in Table S14. Per capita income of the area economy surrounding the protected area, total acreage of the protected area, and the county population surrounding the protected area have a positive effect on non-consumptive visits and is significant at the 10% level. Due to the double log functional form, the per capita income, total acres, and county population coefficients can be interpreted as the percent change in non-consumptive visits. A 1% change in per capita income causes a 1.46% change in non-consumptive visits. A 1% change in total acres causes a 0.46% change in non-consumptive visits. A 1% change in county population causes a 0.26% change in non-consumptive visits. As total acres and county population increase, non-consumptive visits increase at a decreasing rate (diminishing marginal effect).

The coefficient estimates for the model of total hunting visits to the protected areas is shown in Table S15. The presence of a lake has a positive effect on total hunting visits to the protected area and is significant at the 10% level. Total acreage of the protected area has a positive effect on total hunting visits and is significant at the 5% level. Due to the double log functional form, the total acre coefficient can also be interpreted as the percent change in total hunting visits. A 1% change in total acres causes a 0.3% change in total hunting visits. As total acres increase, total hunting visits increase at a decreasing rate (diminishing marginal effect).

The coefficient estimates for the model of total freshwater fishing visits to the protected areas is shown in Table S16. The total acreage of the protected area and the county population surrounding the protected area has a positive effect on fishing visits and is significant at the 5% level. The per capita income of the area economy surrounding the protected area has a negative effect on fishing visits and is significant at the 1% level. Due to the double log functional form, the per capita income, total acres, and county population coefficients can be interpreted as the percent change in fishing visits. A 1% change in total acres causes a 0.49% change in fishing visits. A 1% change in county population causes a 0.65% change in fishing visits. As total acres and county population increase, fishing visits increase at a decreasing rate (diminishing marginal effect). A 1% change in per capita income causes a 4% change in freshwater fishing visits.

A limitation of these visitor use estimating models is that there is no explanatory variable that acknowledges the dependence of the number of visiting days to a protected area on the existing amount of publically available land. A public area attracts more visitors if there are a limited number of existing protected areas. The acreage of pre-existing public land is obtained from the protected areas database [31]. The amount of publicly available land within 60 miles of the acquisition is added to the total acreage of the acquisition, and this acreage is applied to the coefficient on total acreage in the each of the models to calculate the increase in visits. The low and high-end estimates of visits from each of the models are based on the amount of publicly available land within 60 miles that is a viable alternative to the acquisitions.

*7B. Values per trip day for wildlife viewing, total hunting, and freshwater fishing*

Values of fishing, hunting and viewing days come from the recent U.S. Forest Service database and publication by [32]. The completeness of the database for fishing studies is checked by comparing it to the [33] Sport Fishing Database believed to have the most complete coverage of fishing valuation studies. Rosenberger provided a listing of very recent studies up to and including January 2007 that had not been entered into the [32] database. Studies in the database have the most updated values per hunter day, angler day and viewer day tables by geographic region. In addition, all the database studies were disaggregated into three types of fishing (cold, warm, anadromous - i.e., steelhead and salmon), three types of hunting (big game, small game and waterfowl), and two types of viewing (general wildlife viewing and bird viewing). Table S17 indicates the average values per day for hunting, fishing, and wildlife viewing.

**Appendix references**

1. Anderson JR, Hardy EE, Roach JT, Witmer RE (1976) A land use and land cover classification system for use with remote sensor data. U.S. Geological Survey Professional Paper 964. US Geological Survey, Reston, Virginia.

2. Lewis DJ, Plantinga AJ (2007) Policies for habitat fragmentation: Combining econometrics with GIS-based landscape simulations. Land Econ 83:109-127.

3. Lubowski RN, Plantinga AJ, Stavins RN (2006) Land-use change and carbon sinks: econometric estimation of the carbon sequestration supply function. J Environ Econ Manage 51: 135-52.

4. Sundquist ET, Ackerman KV, Bliss NB, Kellndorfer JM, Reeves MC, et al. (2009) Rapid assessment of U.S. forest and soil organic carbon storage and forest biomass carbon sequestration capacity: U.S. Geological Survey Open-File Report, 2009-1283, 15 p., Available: at <http://pubs.er.usgs.gov/usgspubs/ofr/ofr20091283>.

5. Risser P, Birney E, Blocker H, May S, Parton W (1981) The true prairie ecosystem. Hutchinson Ross Pub. Co.[New York]: distributed world wide by Academic Press, Stroudsburg, Pa.

6. Bransby DI, McLaughlin SB, Parrish DJ (1998) A review of carbon and nitrogen balances in switchgrass grown for energy. Biomass Bioenergy **14**:379-384.

7. Oesterheld M, Loreti J, Semmartin M, Paruelo J (1999) Grazing, fire, and climate effects on primary productivity of grasslands and savannas. Ecosyst World:287-306.

8. Zan C, Fyles J, Girouard P, Samson R (2001) Carbon sequestration in perennial bioenergy, annual corn and uncultivated systems in southern Quebec. Agric Ecosyst Environ **86**:135-144.

9. Baer SG, Kitchen DJ, Blair JM, Rice CW (2002) Changes in ecosystem structure and function along a chronosequence of restored grasslands. Ecol Appl **12**:1688-1701.

10. Tilman D, Hill J, Lehman C (2006) Carbon-negative biofuels from low-input high-diversity grassland biomass. Science **314**: 1598-1600.

11. Nelson E, Mendoza G, Regetz J, Polasky S, Tallis H, et al. (2009) Modeling multiple ecosystem services, biodiversity conservation, commodity production, and tradeoffs at landscape scales. Front Ecol Environ 7(1): 4–11.

12. Schuman GE, Reeder JD, Manley JT, Hart RH, Manley WA (1999) Impact of Grazing management on the carbon and nitrogen balance of a mixed-grass rangeland. Ecol Appl 9: 65-71.

13. Intergovernmental Panel on Climate Change (IPCC) (2006) 2006 IPCC Guidelines for
National Greenhouse Gas Inventories. Available: <http://www.ipcc-nggip.iges.or.jp/public/2006gl/>. Accessed 2013 May 3.

14. Fissore C, Baker LA, Hobbie SE, King JY, McFadden JP, Nelson KC, Jakobsdottir I (2011) Carbon, nitrogen, and phosphorus fluxes in household ecosystems in the Minneapolis-Saint Paul, Minnesota, urban region. Ecological Appl 21: 619–639.

15. Smith JE, Heath LS, Skog KE, Birdsey RA (2006) Methods for calculating forest ecosystem and harvested carbon with standard estimates for forest types of the United States. United States Dept. of Agriculture, Forest Service, Northeastern Research Station.

16. Blackard JA, Finco MV, Helmer EH, Holden GR, Hoppus ML, et al. (2008) Mapping U.S. forest biomass using national forest inventory data and moderate resolution information. Remote Sens Environ 112: 1658-1677.

17. Minnesota Department of Transportation (MN DOT). (2009) Statewide road network shapfile for Minnesota.Available: <http://deli.dnr.state.mn.us/metadata.html?id=L300000052102>. Accessed December 2009.

18. Lindenmayer D, Hobbs R, Montague-Drake R, Alexandra J, Bennett A, et al. (2008) A checklist for ecological management of landscapes for conservation. Ecol Lett 11: 78-91.

19. Lubowski RN (2002) Determinants of Land-Use Transitions in the United States: Econometric Analysis of Changes among the Major Land-Use Categories. Ph.D. Dissertation, Harvard University, Cambridge, MA.

20. Lubowski RN, Plantinga AJ, Stavins RN (2008) What drives land-use change in the United States? A national analysis of landowner decisions. Land Econ 84(4): 529-550.

21. Tallis HT, Ricketts T, Guerry AD, Nelson E, Ennaanay D, et al. (2011) InVEST 2.1 beta User’s Guide. The Natural Capital Project, Stanford.

22. USDA-NRCS (2009) Soil Survey Staff, Natural Resources Conservation Service, United States Department of Agriculture. Soil Survey Geographic (SSURGO) Database for Minnesota. Available: http://soildatamart.nrcs.usda.gov.

23. Schenk HJ, Jackson RB (2002) The Global Biogeography of roots. Ecol Monogr 72 (3): 311-328.

24. Allen RG, Pereira LS, Raes D, Smith M (1998) Crop evapotranspiration – guidelines for computing crop water requirements. FAO irrigation and drainage paper 56. Available: <http://www.fao.org/docrep/X0490E/x0490e00.htm#Contents>

25. Zhang L, Dawes WR, Walker GR (2001) Response of mean annual evapotranspiration to vegetation changes at catchment scale. Water Resour Res **37**:701-708.

26. Budyko MI (1974) Climate and Life. Academic: San Diego, California.

27. Reckhow KH, Beaulac MN, Simpson JT (1980) Modeling phosphorus loading and lake response under uncertainty: a manual and compilation of export coefficients. EPA/440/5-80/011. U.S. Environmental Protection Agency, Washington, DC, USA.

28. Azevedo CD, Egan KJ, Herriges JA, Kling CL (2003) The Iowa lakes valuation project: Summary and findings from year one. CARD report.

29. Loomis J, Kroeger T, Richardson L, Casey F (2008) Benefit Transfer and Visitor Use Estimating Model of Wildlife Recreation, Species, and Habitats*.* National Council for Science and the Environment: 2006 Wildlife Habitat Policy Research Program. Available: http://dare.colostate.edu/tools/benefittransfer.aspx

30. Caudill J, Henderson E (2004) Banking on Nature: The economic benefits to local communities of national wildlife refuge visitation. Division of Economics, U.S. Fish and Wildlife Service, Washington, DC.

31. Protected areas database (PAD) – US Technical Working Group. (October 2009) Protected areas database – US Technical Report. Available: <http://www.protectedlands.net/padus/publications.php>. Accessed May 7, 2013.

32. Loomis J. 2005. *Updated outdoor recreation use values on National Forests and other public lands*. PNW-GTR-658, Pac. NW Research Station, USDA Forest Service, Portland, OR. 26 pp.

33. Boyle K, Bishop R, Caudill J, Charbonneau J, Larson D (1998) A Database of Sport Fishing Values. Prepared for U.S. Fish and Wildlife Service. Prepared by Industrial Economics, Cambridge, MA. October 1998.

1. The more spatially detailed Soil Survey Geographic (SSURGO) Database is not currently complete in GIS form for the whole state. [↑](#footnote-ref-1)
