# Supplementary material for: Evaluating the Return in Ecosystem Services from Investment in Public Land Acquisitions
Source: PLoS One. 2013 Jun 11;8(6):e62202. doi: 10.1371/journal.pone.0062202 (PMC3679083; doi:10.1371/journal.pone.0062202)
Supplement: Table S1 — LULC class definitions from the definitions of the grouped classes of the NLCD 1992 used in the maps for Minnesota (from http://www.mrlc.gov/nlcd92_leg.php ). (DOCX) [file pone.0062202.s004.docx]

| **Code** | **Class\ Value** | **Descriptions** |
| --- | --- | --- |
| 1  (NLCD classes 21-23, 85) | Urban | Includes developed open spaces with a mixture of some constructed materials, but mostly vegetation in the form of lawn grasses such as large-lot single-family housing units, parks, golf courses, and vegetation planted in developed settings for recreation, erosion control, or aesthetic purposes. Also included are lands of low, medium, and high intensity with a mixture of constructed materials and vegetation, such as single-family housing units, multifamily housing units, and areas of retail, commercial, and industrial uses. |
| 2  (NLCD classes 61, 82, 83, 84) | Cropland | Includes cultivated crops – Cultivated crops are described as areas used for the production of annual crops, such as corn, soybeans, vegetables, tobacco, and cotton, and also perennial woody crops such as orchards and vineyards. This class also includes all actively tilled land. |
| 3  (NLCD class 81) | Pasture/Hay | Pasture/Hay is described as grasses, legumes, or grass-legume mixtures planted for livestock grazing or the production of seed or hay crops, typically on a perennial cycle. |
| 4  (NLCD classes 41, 42, 43) | Forest | All areas characterized by tree cover (natural or semi-natural woody vegetation, generally greater than 6 meters tall); tree canopy accounts for 25% to 100% of the cover. |
| 5  (NLCD classes 51, 71) | Grassland/Shrub | Areas dominated by upland grasses and forbs. In rare cases, herbaceous cover is less than 25%, but exceeds the combined cover of the woody species present. These areas are not subject to intensive management, but they are often utilized for grazing. Also, areas characterized by natural or semi-natural woody vegetation with aerial stems, generally less than 6 meters tall, with individuals or clumps not touching to interlocking. Both evergreen and deciduous species of true shrubs, young trees, and trees or shrubs that are small or stunted because of environmental conditions are included. |
| 6  (NLCD classes 11, 12) | Water | All areas of open water, generally with less than 25% vegetation or soil cover, and all areas characterized by year-long surface cover of ice and/or snow. |
| 7  (NLCD classes 31-33) | Barren | Areas of bedrock, pavement, scarps, talus, slides, glacial debris, strip mines, gravel pits, and other accumulations of earthen material. Generally, vegetation accounts for less than 15% of total cover. |
| 8  (NLCD classes 91, 92) | Wetlands | Includes woody wetlands and herbaceous wetlands – Areas where forest or shrub land vegetation accounts for greater than 20 percent of vegetative cover and the soil or substrate is periodically saturated with or covered with water. This class also includes areas where perennial herbaceous vegetation accounts for greater than 80 percent of vegetative cover and the soil or substrate is periodically saturated with or covered with water. |
