# Supplementary material for: Evaluating the Return in Ecosystem Services from Investment in Public Land Acquisitions
Source: PLoS One. 2013 Jun 11;8(6):e62202. doi: 10.1371/journal.pone.0062202 (PMC3679083; doi:10.1371/journal.pone.0062202)
Supplement: Table S2 — LULC on the acquisition for the maps with acquisitions, and the maps without acquisitions in 1992, 2022, and 2052 for the baseline and agricultural expansion scenarios. (DOCX) [file pone.0062202.s005.docx]

| **Land use/land cover** | **With acquisitions** | **Without Acquisitions** | | | | |
| --- | --- | --- | --- | --- | --- | --- |
|  |  | **1992** | **Baseline** | | **Agricultural expansion** | |
|  |  |  | **2022** | **2052** | **2022** | **2052** |
| **Urban** | 0 | 1,518 | 2,629 | 3,543 | 2,629 | 3,543 |
| **Cropland** | 0 | 23,960 | 19,180 | 19,801 | 28,733 | 30,793 |
| **Pasture** | 0 | 15,370 | 16,362 | 13,468 | 13,230 | 11,817 |
| **Forest** | 56,102 | 45,085 | 46,795 | 46,896 | 43,748 | 43,371 |
| **Shrub/Grassland** | 38,231 | 8,400 | 9,365 | 10,623 | 5,992 | 4,808 |
| **Water** | 2,605 | 2,605 | 2,605 | 2,605 | 2,605 | 2,605 |
| **Barren** | 46 | 46 | 46 | 46 | 46 | 46 |
| **Wetland** | 26,569 | 26,569 | 26,569 | 26,569 | 26,569 | 26,569 |
| **Total Acres** | 123,552 | 123,552 | 123,552 | 123,552 | 123,552 | 123,552 |
