# Supplementary material for: Evaluating the Return in Ecosystem Services from Investment in Public Land Acquisitions
Source: PLoS One. 2013 Jun 11;8(6):e62202. doi: 10.1371/journal.pone.0062202 (PMC3679083; doi:10.1371/journal.pone.0062202)
Supplement: Table S3 — Distribution of metric tons of stored soil organic carbon (SOC) per hectare within the 30 centimeters of the soil profile by LULC type and county (estimated from [4] ). We assume crop SOC is 75% of natural LULC SOC estimates. (DOCX) [file pone.0062202.s006.docx]

|  | Urban | | Crop | | Pasture | | Forest | | Grassland / shrub | | Water | | Barren | | Wetland | |
| --- | --- | --- | --- | --- | --- | --- | --- | --- | --- | --- | --- | --- | --- | --- | --- | --- |
| County fips | Mean | SD | Mean | SD | Mean | SD | Mean | SD | Mean | SD | Mean | SD | Mean | SD | Mean | SD |
| 27001 | 64 | 89 | 78 | 70 | 86 | 104 | 72 | 98 | 71 | 94 | 149 | 60 | 31 | 65 | 194 | 119 |
| 27003 | 66 | 77 | 89 | 76 | 90 | 90 | 90 | 95 | 120 | 103 | 182 | 61 | 110 | 105 | 185 | 104 |
| 27005 | 70 | 55 | 81 | 59 | 64 | 58 | 97 | 91 | 68 | 75 | 134 | 51 | 41 | 52 | 202 | 83 |
| 27007 | 38 | 68 | 83 | 78 | 52 | 62 | 63 | 84 | 74 | 87 | 124 | 60 | 127 | 150 | 251 | 139 |
| 27009 | 79 | 36 | 79 | 52 | 83 | 48 | 94 | 63 | 104 | 72 | 120 | 44 | 44 | 37 | 162 | 90 |
| 27011 | 91 | 32 | 46 | 22 | 90 | 40 | 47 | 12 | 41 | 21 | 94 | 23 | 84 | 35 | 117 | 34 |
| 27013 | 106 | 42 | 81 | 44 | 100 | 55 | 105 | 52 | 113 | 55 | 96 | 26 | 94 | 43 | 139 | 57 |
| 27015 | 99 | 35 | 55 | 27 | 89 | 42 | 60 | 13 | 71 | 21 | 106 | 34 | 110 | 29 | 117 | 46 |
| 27017 | 49 | 55 | 49 | 47 | 37 | 42 | 40 | 54 | 42 | 51 | 125 | 50 | 31 | 23 | 139 | 102 |
| 27019 | 84 | 58 | 79 | 52 | 102 | 71 | 105 | 79 | 93 | 67 | 132 | 40 | 55 | 26 | 145 | 63 |
| 27021 | 42 | 55 | 67 | 64 | 53 | 60 | 59 | 84 | 66 | 82 | 127 | 59 | 32 | 27 | 183 | 120 |
| 27023 | 114 | 29 | 57 | 28 | 88 | 36 | 53 | 25 | 88 | 32 | 80 | 17 | 102 | 32 | 124 | 27 |
| 27025 | 47 | 53 | 71 | 62 | 64 | 73 | 62 | 79 | 83 | 91 | 133 | 56 | 27 | 43 | 188 | 105 |
| 27027 | 77 | 40 | 71 | 47 | 75 | 42 | 84 | 51 | 115 | 72 | 90 | 34 | 88 | 68 | 119 | 60 |
| 27029 | 60 | 69 | 89 | 85 | 73 | 99 | 102 | 116 | 89 | 110 | 171 | 69 | 93 | 132 | 218 | 122 |
| 27031 | 46 | 26 | 35 | 27 | 44 | 11 | 46 | 28 | 49 | 26 | 28 | 9 | 51 | 15 | 62 | 53 |
| 27033 | 103 | 33 | 77 | 29 | 108 | 46 | 104 | 19 | 99 | 34 | 121 | 34 | 109 | 30 | 139 | 40 |
| 27035 | 45 | 10 | 36 | 16 | 58 | 16 | 56 | 18 | 53 | 18 | 30 | 7 | 42 | 10 | 52 | 20 |
| 27037 | 59 | 47 | 63 | 50 | 89 | 52 | 79 | 62 | 71 | 51 | 96 | 38 | 128 | 63 | 124 | 57 |
| 27039 | 109 | 33 | 55 | 30 | 88 | 35 | 62 | 25 | 49 | 19 | 109 | 36 | 114 | 27 | 137 | 44 |
| 27041 | 75 | 40 | 79 | 53 | 104 | 68 | 103 | 76 | 93 | 69 | 98 | 39 | 48 | 29 | 159 | 80 |
| 27043 | 126 | 26 | 68 | 30 | 122 | 43 | 83 | 21 | 65 | 25 | 144 | 36 | 116 | 30 | 164 | 47 |
| 27045 | 64 | 31 | 33 | 20 | 45 | 23 | 52 | 22 | 27 | 16 | 44 | 14 | 55 | 19 | 58 | 31 |
| 27047 | 105 | 52 | 72 | 42 | 118 | 66 | 97 | 45 | 60 | 35 | 147 | 48 | 100 | 47 | 154 | 70 |
| 27049 | 65 | 31 | 52 | 25 | 63 | 27 | 54 | 21 | 55 | 23 | 84 | 19 | 64 | 22 | 126 | 46 |
| 27051 | 88 | 39 | 56 | 33 | 71 | 44 | 70 | 41 | 72 | 45 | 66 | 24 | 54 | 25 | 102 | 42 |
| 27053 | 63 | 52 | 80 | 53 | 88 | 53 | 101 | 74 | 93 | 59 | 121 | 41 | 78 | 23 | 160 | 73 |
| 27055 | 49 | 22 | 42 | 21 | 39 | 15 | 42 | 20 | 48 | 22 | 74 | 15 | 48 | 26 | 105 | 39 |
| 27057 | 51 | 42 | 63 | 59 | 46 | 32 | 58 | 80 | 49 | 61 | 137 | 67 | 27 | 34 | 192 | 121 |
| 27059 | 40 | 56 | 73 | 66 | 51 | 71 | 59 | 80 | 106 | 100 | 133 | 41 | 95 | 122 | 179 | 87 |
| 27061 | 31 | 48 | 48 | 51 | 35 | 53 | 38 | 65 | 31 | 49 | 115 | 58 | 25 | 33 | 156 | 112 |
| 27063 | 114 | 24 | 74 | 30 | 106 | 37 | 100 | 29 | 100 | 38 | 80 | 16 | 108 | 31 | 135 | 24 |
| 27065 | 60 | 43 | 83 | 56 | 76 | 58 | 91 | 75 | 99 | 76 | 146 | 44 | 42 | 11 | 194 | 89 |
| 27067 | 71 | 52 | 83 | 65 | 110 | 80 | 110 | 89 | 86 | 70 | 112 | 36 | 90 | 51 | 167 | 100 |
| 27069 | 83 | 29 | 73 | 36 | 81 | 42 | 98 | 49 | 89 | 50 | 114 | 44 | 10 | 0 | 144 | 63 |
| 27071 | 86 | 46 | 71 | 40 | 85 | 37 | 84 | 44 | 85 | 41 | 69 | 24 | 68 | 22 | 151 | 73 |
| 27073 | 107 | 26 | 45 | 22 | 91 | 33 | 47 | 20 | 38 | 19 | 73 | 11 | 99 | 30 | 120 | 20 |
| 27073 | 107 | 26 | 45 | 22 | 91 | 33 | 47 | 20 | 38 | 19 | 73 | 11 | 99 | 30 | 120 | 20 |
| 27075 | 43 | 26 | 43 | 30 | 33 | 12 | 52 | 32 | 61 | 39 | 45 | 21 | 41 | 29 | 69 | 55 |
| 27077 | 64 | 51 | 85 | 77 | 62 | 48 | 105 | 109 | 61 | 75 | 188 | 90 | 39 | 48 | 225 | 150 |
| 27079 | 87 | 46 | 75 | 47 | 95 | 63 | 94 | 61 | 97 | 65 | 125 | 42 | 66 | 20 | 141 | 62 |
| 27081 | 104 | 26 | 60 | 23 | 97 | 36 | 33 | 11 | 119 | 30 | 96 | 22 | 89 | 30 | 143 | 32 |
| 27083 | 84 | 45 | 58 | 24 | 94 | 38 | 49 | 11 | 97 | 28 | 96 | 27 | 88 | 39 | 116 | 37 |
| 27085 | 107 | 37 | 82 | 35 | 112 | 47 | 113 | 45 | 109 | 49 | 108 | 36 | 44 | 46 | 140 | 49 |
| 27087 | 102 | 45 | 80 | 73 | 77 | 68 | 104 | 98 | 78 | 86 | 168 | 58 | 99 | 116 | 181 | 108 |
| 27089 | 107 | 35 | 83 | 65 | 96 | 52 | 79 | 55 | 117 | 94 | 145 | 55 | 121 | 89 | 195 | 111 |
| 27091 | 120 | 29 | 84 | 37 | 116 | 45 | 118 | 33 | 116 | 42 | 110 | 32 | 108 | 30 | 143 | 51 |
| 27093 | 84 | 50 | 86 | 45 | 106 | 66 | 120 | 68 | 96 | 68 | 116 | 27 | 16 | 24 | 162 | 59 |
| 27095 | 69 | 53 | 90 | 60 | 92 | 65 | 91 | 76 | 122 | 82 | 132 | 47 | 80 | 0 | 201 | 86 |
| 27097 | 62 | 51 | 84 | 85 | 72 | 81 | 93 | 115 | 86 | 108 | 118 | 58 | 30 | 37 | 205 | 155 |
| 27099 | 77 | 41 | 49 | 41 | 88 | 56 | 62 | 37 | 49 | 33 | 69 | 32 | 86 | 47 | 105 | 84 |
| 27101 | 113 | 23 | 77 | 29 | 110 | 38 | 104 | 30 | 109 | 31 | 88 | 20 | 101 | 37 | 138 | 26 |
| 27103 | 81 | 45 | 65 | 36 | 74 | 44 | 82 | 37 | 74 | 43 | 106 | 29 | 94 | 47 | 126 | 49 |
| 27105 | 119 | 21 | 80 | 32 | 117 | 30 | 109 | 26 | 112 | 28 | 114 | 38 | 103 | 22 | 143 | 54 |
| 27107 | 97 | 30 | 65 | 36 | 77 | 39 | 95 | 41 | 88 | 45 | 80 | 25 | 54 | 34 | 105 | 59 |
| 27109 | 73 | 35 | 40 | 23 | 66 | 37 | 55 | 26 | 37 | 21 | 68 | 23 | 59 | 26 | 76 | 36 |
| 27111 | 64 | 48 | 93 | 61 | 79 | 55 | 106 | 85 | 106 | 85 | 129 | 50 | 34 | 38 | 212 | 93 |
| 27113 | 89 | 40 | 92 | 61 | 125 | 76 | 135 | 79 | 142 | 90 | 152 | 48 | 68 | 83 | 133 | 85 |
| 27115 | 69 | 39 | 59 | 48 | 76 | 46 | 78 | 63 | 78 | 54 | 70 | 30 | 68 | 18 | 106 | 75 |
| 27117 | 99 | 16 | 60 | 23 | 105 | 30 | 55 | 15 | 105 | 30 | 78 | 18 | 85 | 17 | 127 | 28 |
| 27119 | 100 | 33 | 77 | 58 | 92 | 78 | 107 | 76 | 107 | 91 | 113 | 49 | 31 | 34 | 133 | 90 |
| 27121 | 66 | 64 | 82 | 68 | 90 | 79 | 110 | 102 | 83 | 81 | 151 | 62 | 49 | 34 | 163 | 107 |
| 27123 | 35 | 49 | 65 | 67 | 65 | 88 | 71 | 94 | 72 | 91 | 145 | 65 | 27 | 12 | 144 | 114 |
| 27125 | 78 | 31 | 62 | 33 | 107 | 60 | 70 | 35 | 104 | 59 | 76 | 28 | 9 | 21 | 112 | 58 |
| 27127 | 110 | 24 | 60 | 29 | 96 | 44 | 62 | 21 | 103 | 31 | 69 | 21 | 105 | 31 | 116 | 38 |
| 27129 | 118 | 32 | 65 | 39 | 95 | 47 | 79 | 41 | 100 | 45 | 113 | 40 | 103 | 56 | 116 | 51 |
| 27131 | 60 | 47 | 72 | 51 | 92 | 73 | 93 | 75 | 92 | 70 | 136 | 43 | 53 | 45 | 137 | 62 |
| 27133 | 114 | 23 | 67 | 23 | 113 | 29 | 73 | 15 | 115 | 27 | 68 | 10 | 106 | 24 | 127 | 21 |
| 27135 | 89 | 42 | 73 | 59 | 92 | 55 | 81 | 72 | 89 | 84 | 147 | 50 | 44 | 45 | 161 | 97 |
| 27137 | 49 | 43 | 53 | 42 | 82 | 47 | 59 | 47 | 63 | 45 | 49 | 25 | 57 | 48 | 119 | 79 |
| 27139 | 78 | 90 | 82 | 80 | 87 | 107 | 93 | 116 | 83 | 105 | 191 | 83 | 50 | 74 | 168 | 121 |
| 27141 | 68 | 52 | 69 | 63 | 69 | 69 | 69 | 80 | 92 | 99 | 144 | 40 | 32 | 26 | 150 | 98 |
| 27143 | 119 | 38 | 75 | 45 | 127 | 60 | 111 | 48 | 91 | 47 | 104 | 24 | 125 | 59 | 127 | 57 |
| 27145 | 68 | 54 | 89 | 61 | 93 | 72 | 108 | 87 | 92 | 80 | 116 | 46 | 50 | 24 | 189 | 89 |
| 27147 | 102 | 51 | 77 | 49 | 116 | 83 | 111 | 67 | 55 | 40 | 141 | 49 | 88 | 61 | 170 | 77 |
| 27149 | 98 | 33 | 71 | 46 | 89 | 45 | 103 | 49 | 88 | 61 | 135 | 51 | 102 | 37 | 118 | 59 |
| 27151 | 118 | 38 | 85 | 41 | 104 | 48 | 124 | 50 | 113 | 48 | 93 | 20 | 119 | 41 | 138 | 34 |
| 27153 | 76 | 52 | 86 | 58 | 85 | 58 | 103 | 81 | 100 | 78 | 109 | 44 | 55 | 24 | 182 | 83 |
| 27155 | 110 | 23 | 39 | 25 | 91 | 41 | 29 | 15 | 38 | 23 | 110 | 32 | 104 | 26 | 115 | 34 |
| 27157 | 62 | 19 | 49 | 20 | 52 | 13 | 54 | 16 | 59 | 23 | 76 | 21 | 40 | 28 | 110 | 40 |
| 27159 | 70 | 50 | 80 | 65 | 93 | 68 | 102 | 89 | 103 | 89 | 135 | 55 | 28 | 20 | 154 | 110 |
| 27161 | 119 | 37 | 86 | 49 | 112 | 62 | 113 | 65 | 111 | 61 | 125 | 24 | 118 | 20 | 166 | 42 |
| 27163 | 46 | 53 | 55 | 55 | 53 | 58 | 52 | 61 | 55 | 68 | 105 | 50 | 54 | 21 | 135 | 108 |
| 27165 | 113 | 35 | 45 | 27 | 99 | 46 | 34 | 13 | 53 | 25 | 124 | 29 | 97 | 35 | 123 | 53 |
| 27167 | 117 | 28 | 87 | 45 | 109 | 60 | 98 | 31 | 169 | 84 | 80 | 15 | 112 | 27 | 139 | 54 |
| 27169 | 44 | 39 | 45 | 23 | 46 | 17 | 43 | 15 | 45 | 17 | 92 | 24 | 44 | 18 | 102 | 58 |
| 27171 | 80 | 43 | 74 | 55 | 84 | 62 | 93 | 75 | 89 | 73 | 132 | 40 | 49 | 27 | 150 | 81 |
| 27173 | 92 | 30 | 63 | 26 | 93 | 34 | 72 | 22 | 95 | 33 | 69 | 17 | 90 | 32 | 116 | 28 |
