# Supplementary material for: Evaluating the Return in Ecosystem Services from Investment in Public Land Acquisitions
Source: PLoS One. 2013 Jun 11;8(6):e62202. doi: 10.1371/journal.pone.0062202 (PMC3679083; doi:10.1371/journal.pone.0062202)
Supplement: Table S4 — Metric tons of stored biomass carbon per hectare by non-forest LULC type. (DOCX) [file pone.0062202.s007.docx]

| **LULC** | **Biomass**  **Mg ha^-1^**  **Mean (SD)** | **Number of estimates** | **Notes** | **Source** |
| --- | --- | --- | --- | --- |
| Grassland | 11.88 (1.90) | 10 | Equilibrium achieved at 50 years. Belowground biomass is the only source of biomass carbon considered. | [5], [6], [7], [8], [9], [10], [11] |
| Agriculture | 4.79  (2.30) | 6 | Equilibrium achieved at 20 years. Belowground biomass is the only source of biomass carbon considered. Pastures are continuously grazed at 2 head per hectare. Hayfields assumed to be 50% of natural grassland. | [12], [13] |
| Urban | 17.30 | 1 | Equilibrium achieved at 50 years. | [14] |
