# Supplementary material for: Evaluating the Return in Ecosystem Services from Investment in Public Land Acquisitions
Source: PLoS One. 2013 Jun 11;8(6):e62202. doi: 10.1371/journal.pone.0062202 (PMC3679083; doi:10.1371/journal.pone.0062202)
Supplement: Table S6 — Metric tons of stored biomass carbon per hectare on private forests and rotation length (in years) using estimates in [16] . (DOCX) [file pone.0062202.s009.docx]

| **County FIPs** | **Metric tons / ha** | **Rotation in years** |
| --- | --- | --- |
| 27001 | 46.1 | 25.7 |
| 27003 | 49.9 | 35.0 |
| 27005 | 46.8 | 27.2 |
| 27007 | 37.5 | 26.3 |
| 27009 | 50.4 | 34.1 |
| 27011 | 56.1 | 31.7 |
| 27013 | 59.7 | 35.0 |
| 27015 | 59.7 | 35.0 |
| 27017 | 44.3 | 25.5 |
| 27019 | 55.9 | 31.7 |
| 27021 | 49.2 | 27.1 |
| 27023 | 57.9 | 31.7 |
| 27025 | 52.1 | 30.9 |
| 27027 | 48.6 | 33.0 |
| 27029 | 46.6 | 26.1 |
| 27031 | 46.4 | 25.9 |
| 27033 | 55.9 | 31.7 |
| 27035 | 51.6 | 28.8 |
| 27037 | 56.7 | 33.4 |
| 27039 | 57.5 | 35.0 |
| 27041 | 51.0 | 31.7 |
| 27043 | 60.9 | 28.3 |
| 27045 | 58.1 | 33.9 |
| 27047 | 56.4 | 35.0 |
| 27049 | 61.7 | 31.9 |
| 27051 | 51.9 | 35.0 |
| 27053 | 55.6 | 35.0 |
| 27055 | 60.9 | 34.2 |
| 27057 | 45.9 | 28.4 |
| 27059 | 50.1 | 31.4 |
| 27061 | 43.0 | 26.0 |
| 27063 | 60.1 | 31.7 |
| 27065 | 54.6 | 27.8 |
| 27067 | 54.7 | 31.7 |
| 27069 | 37.8 | 25.3 |
| 27071 | 34.0 | 25.6 |
| 27073 | 54.1 | 31.7 |
| 27075 | 45.1 | 25.6 |
| 27077 | 30.6 | 26.1 |
| 27079 | 55.2 | 31.7 |
| 27081 | 52.1 | 31.7 |
| 27083 | 53.8 | 31.7 |
| 27085 | 54.9 | 31.7 |
| 27087 | 49.0 | 26.6 |
| 27089 | 38.6 | 25.4 |
| 27091 | 60.3 | 35.0 |
| 27093 | 52.8 | 31.7 |
| 27095 | 53.7 | 27.8 |
| 27097 | 52.0 | 29.5 |
| 27099 | 58.1 | 35.0 |
| 27101 | 53.6 | 31.7 |
| 27103 | 57.9 | 31.7 |
| 27105 | 0.0 | 31.7 |
| 27107 | 47.4 | 25.8 |
| 27109 | 58.0 | 34.4 |
| 27111 | 49.4 | 29.2 |
| 27113 | 43.6 | 27.1 |
| 27115 | 52.3 | 26.1 |
| 27117 | 45.1 | 31.7 |
| 27119 | 45.4 | 27.9 |
| 27121 | 51.9 | 35.0 |
| 27123 | 53.0 | 31.7 |
| 27125 | 46.0 | 25.0 |
| 27127 | 57.2 | 35.0 |
| 27129 | 57.8 | 35.0 |
| 27131 | 58.3 | 35.0 |
| 27133 | 48.4 | 31.7 |
| 27135 | 36.2 | 26.2 |
| 27137 | 42.5 | 26.0 |
| 27139 | 56.0 | 29.8 |
| 27141 | 46.8 | 34.4 |
| 27143 | 53.6 | 35.0 |
| 27145 | 52.9 | 34.3 |
| 27147 | 56.7 | 35.0 |
| 27149 | 48.7 | 31.7 |
| 27151 | 52.1 | 31.7 |
| 27153 | 49.7 | 29.9 |
| 27155 | 57.0 | 31.7 |
| 27157 | 61.8 | 33.8 |
| 27159 | 38.7 | 30.9 |
| 27161 | 58.5 | 35.0 |
| 27163 | 55.7 | 31.7 |
| 27165 | 61.2 | 31.7 |
| 27167 | 47.0 | 31.7 |
| 27169 | 63.2 | 34.9 |
| 27171 | 53.1 | 31.3 |
| 27173 | 52.7 | 35.0 |
