# Supplementary material for: Evaluating the Return in Ecosystem Services from Investment in Public Land Acquisitions
Source: PLoS One. 2013 Jun 11;8(6):e62202. doi: 10.1371/journal.pone.0062202 (PMC3679083; doi:10.1371/journal.pone.0062202)
Supplement: Table S7 — Metric tons of stored biomass carbon per hectare in 2052 on forests conserved in 1992 assuming the 1992 LULC was a private forest using “B” tables in [15] . (DOCX) [file pone.0062202.s010.docx]

| **County FIPs** | **Mg / ha** | **Stand Age** |
| --- | --- | --- |
| 27001 | 112.95 | 72.85 |
| 27003 | 138.87 | 77.50 |
| 27005 | 119.91 | 73.62 |
| 27007 | 117.95 | 73.17 |
| 27009 | 145.47 | 77.04 |
| 27011 | 139.78 | 75.83 |
| 27013 | 138.87 | 77.50 |
| 27015 | 138.87 | 77.50 |
| 27017 | 112.74 | 72.74 |
| 27019 | 139.78 | 75.83 |
| 27021 | 120.28 | 73.54 |
| 27023 | 139.78 | 75.83 |
| 27025 | 127.33 | 75.47 |
| 27027 | 133.24 | 76.51 |
| 27029 | 115.93 | 73.03 |
| 27031 | 116.04 | 72.97 |
| 27033 | 139.78 | 75.83 |
| 27035 | 124.36 | 74.40 |
| 27037 | 134.32 | 76.70 |
| 27039 | 138.87 | 77.50 |
| 27041 | 139.78 | 75.83 |
| 27043 | 119.85 | 74.15 |
| 27045 | 135.75 | 76.95 |
| 27047 | 138.87 | 77.50 |
| 27049 | 129.99 | 75.94 |
| 27051 | 138.87 | 77.50 |
| 27053 | 138.87 | 77.50 |
| 27055 | 136.49 | 77.08 |
| 27057 | 129.17 | 74.19 |
| 27059 | 131.81 | 75.72 |
| 27061 | 116.25 | 73.02 |
| 27063 | 139.78 | 75.83 |
| 27065 | 118.31 | 73.88 |
| 27067 | 139.78 | 75.83 |
| 27069 | 111.23 | 72.64 |
| 27071 | 114.12 | 72.81 |
| 27073 | 139.78 | 75.83 |
| 27075 | 114.25 | 72.82 |
| 27077 | 117.12 | 73.06 |
| 27079 | 139.78 | 75.83 |
| 27081 | 139.78 | 75.83 |
| 27083 | 139.78 | 75.83 |
| 27085 | 139.78 | 75.83 |
| 27087 | 115.54 | 73.29 |
| 27089 | 111.50 | 72.68 |
| 27091 | 138.87 | 77.50 |
| 27093 | 139.78 | 75.83 |
| 27095 | 118.43 | 73.90 |
| 27097 | 124.46 | 74.74 |
| 27099 | 138.87 | 77.50 |
| 27101 | 139.78 | 75.83 |
| 27103 | 139.78 | 75.83 |
| 27105 | 139.78 | 75.83 |
| 27107 | 112.87 | 72.92 |
| 27109 | 137.30 | 77.22 |
| 27111 | 123.25 | 74.62 |
| 27113 | 116.37 | 73.54 |
| 27115 | 115.18 | 73.07 |
| 27117 | 139.78 | 75.83 |
| 27119 | 118.72 | 73.95 |
| 27121 | 138.87 | 77.50 |
| 27123 | 139.78 | 75.83 |
| 27125 | 110.45 | 72.50 |
| 27127 | 138.87 | 77.50 |
| 27129 | 138.87 | 77.50 |
| 27131 | 138.87 | 77.50 |
| 27133 | 139.78 | 75.83 |
| 27135 | 117.34 | 73.11 |
| 27137 | 116.70 | 73.02 |
| 27139 | 124.20 | 74.92 |
| 27141 | 139.84 | 77.22 |
| 27143 | 138.87 | 77.50 |
| 27145 | 136.95 | 77.16 |
| 27147 | 138.87 | 77.50 |
| 27149 | 139.78 | 75.83 |
| 27151 | 139.78 | 75.83 |
| 27153 | 125.01 | 74.93 |
| 27155 | 139.78 | 75.83 |
| 27157 | 135.57 | 76.92 |
| 27159 | 140.83 | 75.45 |
| 27161 | 138.87 | 77.50 |
| 27163 | 139.78 | 75.83 |
| 27165 | 139.78 | 75.83 |
| 27167 | 139.78 | 75.83 |
| 27169 | 138.50 | 77.43 |
| 27171 | 128.21 | 75.63 |
| 27173 | 138.87 | 77.50 |

on t aerubvs should refernce these numbers.rom but these are the numbers I calcauated two weeks ago.ro-agriculture scenario"
