# Supplementary material for: Evaluating the Return in Ecosystem Services from Investment in Public Land Acquisitions
Source: PLoS One. 2013 Jun 11;8(6):e62202. doi: 10.1371/journal.pone.0062202 (PMC3679083; doi:10.1371/journal.pone.0062202)
Supplement: Table S8 — Metric tons of stored biomass carbon per hectare in 2052 on forests conserved in 1992 assuming the 1992 LULC was a private non-forest using “B” tables in [15] . (DOCX) [file pone.0062202.s011.docx]

| **County FIPs** | **Mg / ha** | **Stand Age** |
| --- | --- | --- |
| 27001 | 89.33 | 60 |
| 27003 | 103.29 | 60 |
| 27005 | 94.34 | 60 |
| 27007 | 93.54 | 60 |
| 27009 | 111.22 | 60 |
| 27011 | 108.64 | 60 |
| 27013 | 103.29 | 60 |
| 27015 | 103.29 | 60 |
| 27017 | 89.39 | 60 |
| 27019 | 108.64 | 60 |
| 27021 | 94.94 | 60 |
| 27023 | 108.64 | 60 |
| 27025 | 96.97 | 60 |
| 27027 | 100.20 | 60 |
| 27029 | 91.88 | 60 |
| 27031 | 92.14 | 60 |
| 27033 | 108.64 | 60 |
| 27035 | 96.80 | 60 |
| 27037 | 100.80 | 60 |
| 27039 | 103.29 | 60 |
| 27041 | 108.64 | 60 |
| 27043 | 92.87 | 60 |
| 27045 | 101.58 | 60 |
| 27047 | 103.29 | 60 |
| 27049 | 98.43 | 60 |
| 27051 | 103.29 | 60 |
| 27053 | 103.29 | 60 |
| 27055 | 101.98 | 60 |
| 27057 | 102.24 | 60 |
| 27059 | 100.85 | 60 |
| 27061 | 92.23 | 60 |
| 27063 | 108.64 | 60 |
| 27065 | 92.03 | 60 |
| 27067 | 108.64 | 60 |
| 27069 | 88.15 | 60 |
| 27071 | 90.62 | 60 |
| 27073 | 108.64 | 60 |
| 27075 | 90.73 | 60 |
| 27077 | 93.01 | 60 |
| 27079 | 108.64 | 60 |
| 27081 | 108.64 | 60 |
| 27083 | 108.64 | 60 |
| 27085 | 108.64 | 60 |
| 27087 | 90.77 | 60 |
| 27089 | 88.29 | 60 |
| 27091 | 103.29 | 60 |
| 27093 | 108.64 | 60 |
| 27095 | 92.09 | 60 |
| 27097 | 96.00 | 60 |
| 27099 | 103.29 | 60 |
| 27101 | 108.64 | 60 |
| 27103 | 108.64 | 60 |
| 27105 | 108.64 | 60 |
| 27107 | 89.04 | 60 |
| 27109 | 102.43 | 60 |
| 27111 | 95.09 | 60 |
| 27113 | 90.96 | 60 |
| 27115 | 91.01 | 60 |
| 27117 | 108.64 | 60 |
| 27119 | 92.25 | 60 |
| 27121 | 103.29 | 60 |
| 27123 | 108.64 | 60 |
| 27125 | 87.72 | 60 |
| 27127 | 103.29 | 60 |
| 27129 | 103.29 | 60 |
| 27131 | 103.29 | 60 |
| 27133 | 108.64 | 60 |
| 27135 | 93.09 | 60 |
| 27137 | 92.67 | 60 |
| 27139 | 95.25 | 60 |
| 27141 | 105.02 | 60 |
| 27143 | 103.29 | 60 |
| 27145 | 102.24 | 60 |
| 27147 | 103.29 | 60 |
| 27149 | 108.64 | 60 |
| 27151 | 108.64 | 60 |
| 27153 | 96.05 | 60 |
| 27155 | 108.64 | 60 |
| 27157 | 101.48 | 60 |
| 27159 | 110.73 | 60 |
| 27161 | 103.29 | 60 |
| 27163 | 108.64 | 60 |
| 27165 | 108.64 | 60 |
| 27167 | 108.64 | 60 |
| 27169 | 103.08 | 60 |
| 27171 | 97.45 | 60 |
| 27173 | 103.29 | 60 |
