# Supplementary material for: Evaluating the Return in Ecosystem Services from Investment in Public Land Acquisitions
Source: PLoS One. 2013 Jun 11;8(6):e62202. doi: 10.1371/journal.pone.0062202 (PMC3679083; doi:10.1371/journal.pone.0062202)
Supplement: Table S9 — Metric tons of stored soil and biomass carbon in 1992 and the carbon stored with and without conservation in 2037 and 2052 for each LULC change scenario. (DOCX) [file pone.0062202.s012.docx]

| \| **Mg** \|  \| **Without conservation** \| \| \| \| **With conservation** \| \| \| --- \| --- \| --- \| --- \| --- \| --- \| --- \| --- \| \|  \|  \| **Baseline** \| \| **Ag development** \| \| \|  \| **1992** \| **2037** \| **2052** \| **2037** \| **2052** \| **2037** \| **2052** \| \| Soil C \| 4,375,968 \| 4,400,910 \| 4,410,174 \| 4,356,221 \| 4,341,686 \| 4,532,788 \| 4,550,212 \| \|  \|  \|  \|  \|  \|  \|  \|  \| \| Biomass C – [15] \| 581,187 \| 585,169 \| 592,327 \| 556,023 \| 551,830 \| -- \| 2,825,672 \| \| Biomass C – [16] \| 990,591 \| 1,007,192 \| 1,014,951 \| 952,558 \| 944,807 \| -- \| 2,825,672 \| \|  \|  \|  \|  \|  \|  \|  \|  \| \| Total C \|  \|  \|  \|  \|  \|  \|  \| \| Low estimate \| 3,148,599 \| 3,142,056 \| 3,166,111 \| 3,098,817 \| 3,087,663 \| -- \| 7,375,884 \| \| High estimate \| 5,366,559 \| 5,408,102 \| 5,425,125 \| 5,308,778 \| 5,286,493 \| -- \| 7,375,884 \| |
| --- | --- | --- | --- | --- | --- | --- | --- | --- | --- | --- | --- | --- | --- | --- | --- | --- | --- | --- | --- | --- | --- | --- | --- | --- | --- | --- | --- | --- | --- | --- | --- | --- | --- | --- | --- | --- | --- | --- | --- | --- | --- | --- | --- | --- | --- | --- | --- | --- | --- | --- | --- | --- | --- | --- | --- | --- | --- | --- | --- | --- | --- | --- | --- | --- | --- | --- | --- | --- | --- | --- | --- | --- | --- | --- | --- | --- | --- | --- | --- | --- | --- | --- | --- | --- | --- | --- |
