# Supplementary material for: Evaluating the Return in Ecosystem Services from Investment in Public Land Acquisitions
Source: PLoS One. 2013 Jun 11;8(6):e62202. doi: 10.1371/journal.pone.0062202 (PMC3679083; doi:10.1371/journal.pone.0062202)
Supplement: Table S10 — Sensitivity to degradation sources and habitat suitability weights each LULC type for breeding bird biodiversity. Higher numbers indicate more sensitivity or more suitable habitat. (DOCX) [file pone.0062202.s013.docx]

| **LULC** | **Agriculture area** | **Urban area** | **Primary roads** | **Secondary roads** | **Light roads** | **Habitat Suitability** |
| --- | --- | --- | --- | --- | --- | --- |
| Open water | 0.00 | 0.00 | 0.00 | 0.00 | 0.00 | 0.00 |
| Urban | 0.00 | 0.00 | 0.00 | 0.00 | 0.00 | 0.00 |
| Barren | 0.00 | 0.00 | 0.00 | 0.00 | 0.00 | 0.00 |
| Forest | 0.70 | 0.80 | 0.80 | 0.60 | 0.40 | 1.00 |
| Grassland | 0.60 | 0.70 | 0.70 | 0.50 | 0.40 | 1.00 |
| Agriculture | 0.00 | 0.50 | 0.50 | 0.40 | 0.40 | 0.20 |
| Wetland | 0.60 | 0.80 | 0.80 | 0.60 | 0.40 | 1.00 |
