# Supplementary material for: Evaluating the Return in Ecosystem Services from Investment in Public Land Acquisitions
Source: PLoS One. 2013 Jun 11;8(6):e62202. doi: 10.1371/journal.pone.0062202 (PMC3679083; doi:10.1371/journal.pone.0062202)
Supplement: Table S11 — Weights and effective distances for degradation sources used in the habitat quality model. (DOCX) [file pone.0062202.s014.docx]

| **Degradation source** | **Maximum effective distance of degradation source (km)** | **Weight** |
| --- | --- | --- |
| Agriculture area | 4.0 | 0.8 |
| Urban area | 5.0 | 1.0 |
| Primary roads | 3.0 | 0.8 |
| Secondary roads | 2.0 | 0.7 |
| Light roads | 1.0 | 0.5 |
