# Supplementary material for: Evaluating the Return in Ecosystem Services from Investment in Public Land Acquisitions
Source: PLoS One. 2013 Jun 11;8(6):e62202. doi: 10.1371/journal.pone.0062202 (PMC3679083; doi:10.1371/journal.pone.0062202)
Supplement: Table S12 — Average per acre net returns to managed forestry from [3] , [19] , [20] (all values are expressed in 1992 dollars; 1992 = 100). (DOCX) [file pone.0062202.s015.docx]

| **County FIPS Code** | **1992 Managed Forestry** | | **County FIPS Code** | **1992 Managed Forestry** | **County FIPS Code** | | **1992 Managed Forestry** | |
| --- | --- | --- | --- | --- | --- | --- | --- | --- |
| 27001 | -$0.31 | | 27031 | $0.45 | 27061 | | $0.46 | |
| 27003 | $2.36 | | 27033 | $0.83 | 27063 | | $0.83 | |
| 27005 | $0.89 | | 27035 | $1.37 | 27065 | | $0.17 | |
| 27007 | $0.79 | | 27037 | $1.88 | 27067 | | $0.83 | |
| 27009 | $4.71 | | 27039 | $2.36 | 27069 | | -$0.59 | |
| 27011 | $0.83 | | 27041 | $0.83 | 27071 | | $0.06 | |
| 27013 | $2.36 | | 27043 | $0.33 | 27073 | | $0.83 | |
| 27015 | $2.36 | | 27045 | $2.03 | 27075 | | $0.09 | |
| 27017 | -$0.27 | | 27047 | $2.36 | 27077 | | $0.66 | |
| 27019 | $0.83 | | 27049 | $1.41 | 27079 | | $0.83 | |
| 27021 | $1.08 | | 27051 | $2.36 | 27081 | | $0.83 | |
| 27023 | $0.83 | | 27053 | $2.36 | 27083 | | $0.83 | |
| 27025 | $1.13 | | 27055 | $2.11 | 27085 | | $0.83 | |
| 27027 | $1.76 | | 27057 | $2.95 | 27087 | | -$0.03 | |
| 27029 | $0.36 | | 27059 | $2.15 | 27089 | | -$0.56 | |
| 27091 | $2.36 | 27133 | | $0.83 | |  | |  |
| 27093 | $0.83 | 27135 | | $0.67 | |  | |  |
| 27095 | $0.18 | 27137 | | $0.58 | |  | |  |
| 27097 | $1.06 | 27139 | | $0.80 | |  | |  |
| 27099 | $2.36 | 27141 | | $2.92 | |  | |  |
| 27101 | $0.83 | 27143 | | $2.36 | |  | |  |
| 27103 | $0.83 | 27145 | | $2.16 | |  | |  |
| 27105 | $0.83 | 27147 | | $2.36 | |  | |  |
| 27107 | -$0.41 | 27149 | | $0.83 | |  | |  |
| 27109 | $2.19 | 27151 | | $0.83 | |  | |  |
| 27111 | $0.83 | 27153 | | $1.02 | |  | |  |
| 27113 | -$0.04 | 27155 | | $0.83 | |  | |  |
| 27115 | $0.10 | 27157 | | $2.01 | |  | |  |
| 27117 | $0.83 | 27159 | | $5.00 | |  | |  |
| 27119 | $0.21 | 27161 | | $2.36 | |  | |  |
| 27121 | $2.36 | 27163 | | $0.83 | |  | |  |
| 27123 | $0.83 | 27165 | | $0.83 | |  | |  |
| 27125 | -$0.67 | 27167 | | $0.83 | |  | |  |
| 27127 | $2.36 | 27169 | | $2.32 | |  | |  |
| 27129 | $2.36 | 27171 | | $1.22 | |  | |  |
| 27131 | $2.36 | 27173 | | $2.36 | |  | |  |
