# Supplementary material for: Evaluating the Return in Ecosystem Services from Investment in Public Land Acquisitions
Source: PLoS One. 2013 Jun 11;8(6):e62202. doi: 10.1371/journal.pone.0062202 (PMC3679083; doi:10.1371/journal.pone.0062202)
Supplement: Table S13 — Estimates for nutrient loading, evapotranspiration, rooting depth, available water capacity, and vegetation filtering. (DOCX) [file pone.0062202.s016.docx]

| **LULC** | **Evapotranspiration** | **Rooting**  **depth** | **Phosphorous**  **loading** | **Phosphorous**  **filtering** |
| --- | --- | --- | --- | --- |
| Open Water | 542 | 1 | 1 | 0 |
| Urban | 1 | 1 | 1910 | 0 |
| Barren | 1 | 1 | 118 | 5 |
| Forest | 1000 | 104 | 236 | 60 |
| Grassland | 750 | 91 | 100 | 50 |
| Agriculture | 500 | 64 | 4460 | 40 |
| Pasture | 750 | 91 | 2755 | 50 |
| Wetland | 400 | 1 | 1 | 80 |

Source: Reckhow et al 1980, Allen et al. 1998, Schenk and Jackson 2002.
