# Supplementary material for: Evaluating the Return in Ecosystem Services from Investment in Public Land Acquisitions
Source: PLoS One. 2013 Jun 11;8(6):e62202. doi: 10.1371/journal.pone.0062202 (PMC3679083; doi:10.1371/journal.pone.0062202)
Supplement: Table S14 — Coefficient estimates for the model of non-consumptive (wildlife viewing) visits. (DOCX) [file pone.0062202.s017.docx]

| Variable | Coefficient | Std. Error | t-Statistic | Prob. |
| --- | --- | --- | --- | --- |
| Constant | -12.11 | 8.32 | -1.45 | 0.14 |
| Ln Per Capita Income | 1.45 | 0.80 | 1.81 | 0.07 |
| Ln Total Acres | 0.46 | 0.12 | 3.69 | 0.00 |
| Ln County Population | 0.25 | 0.14 | 1.74 | 0.08 |

Number of observations: 87. Adjusted R-squared is 0.21.
