# Supplementary material for: Evaluating the Return in Ecosystem Services from Investment in Public Land Acquisitions
Source: PLoS One. 2013 Jun 11;8(6):e62202. doi: 10.1371/journal.pone.0062202 (PMC3679083; doi:10.1371/journal.pone.0062202)
Supplement: Table S15 — Coefficient estimates for the model of total hunting visits. (DOCX) [file pone.0062202.s018.docx]

| Variable | Coefficient | Std. Error | t-Statistic | Prob. |
| --- | --- | --- | --- | --- |
| Constant | 3.96 | 1.47 | 2.69 | 0.00 |
| Lake | 0.94 | 0.51 | 1.82 | 0.07 |
| Ln Total Acres | 0.30 | 0.14 | 2.12 | 0.03 |

Number of observations: 73. Adjusted R-squared is 0.08.
