# Supplementary material for: Evaluating the Return in Ecosystem Services from Investment in Public Land Acquisitions
Source: PLoS One. 2013 Jun 11;8(6):e62202. doi: 10.1371/journal.pone.0062202 (PMC3679083; doi:10.1371/journal.pone.0062202)
Supplement: Table S16 — Coefficient estimates for the model of total freshwater fishing visits. (DOCX) [file pone.0062202.s019.docx]

| Variable | Coefficient | Std. Error | t-Statistic | Prob. |
| --- | --- | --- | --- | --- |
| Constant | 35.75 | 15.74 | 2.27 | 0.02 |
| Ln Total Acres | 0.49 | 0.21 | 2.24 | 0.02 |
| Ln Per Capita Income | -4.04 | 1.50 | -2.67 | 0.00 |
| Ln County Population | 0.65 | 0.23 | 2.81 | 0.00 |

Number of observations: 62. Adjusted R-squared is 0.23.
