# Supplementary material for: Evaluating the Return in Ecosystem Services from Investment in Public Land Acquisitions
Source: PLoS One. 2013 Jun 11;8(6):e62202. doi: 10.1371/journal.pone.0062202 (PMC3679083; doi:10.1371/journal.pone.0062202)
Supplement: Table S17 — Average values per day for hunting, fishing, and wildlife viewing. (DOCX) [file pone.0062202.s020.docx]

| Species category | Average value per day for the Northeast | Number of estimates | Number of studies |
| --- | --- | --- | --- |
| Hunting |  |  | 21 |
| Big game | 58.45 | 142 |  |
| Small game | 32.40 | 11 |  |
| Waterfowl | 35.99 | 39 |  |
| All game | 42.28 | 192 |  |
| Fishing |  |  |  |
| Cold water | 39.54 | 58 | 14 |
| Wildlife viewing | 46.48 | 88 | 9 |

Values are reported in 2010$.
